# Formation of DNA Adducts by 1-Methoxy-3-indolylmethylalcohol, a Breakdown Product of a Glucosinolate, in the Mouse: Impact of the SULT1A1 Status—Wild-Type, Knockout or Humanised

**DOI:** 10.3390/ijms25073824

**Published:** 2024-03-29

**Authors:** Hansruedi Glatt, Sarah Yasmin Weißenberg, Anke Ehlers, Alfonso Lampen, Albrecht Seidel, Fabian Schumacher, Wolfram Engst, Walter Meinl

**Affiliations:** 1 Department Food Safety, Federal Institute of Risk Assessment (BfR), Max-Dohrn-Strasse 8–10, 10589 Berlin, Germany; sarah.weissenberg@live.com (S.Y.W.); anke.ehlers@bfr.bund.de (A.E.); alfonso.lampen@bfr.bund.de (A.L.); 2 Department of Nutritional Toxicology, German Institute of Human Nutrition (DIfE), Potsdam-Rehbrücke, Arthur-Scheunert-Allee 114–116, 14558 Nuthetal, Germany; fabian.schumacher@fu-berlin.de (F.S.); wolfram.engst@web.de (W.E.); meinl@dife.de (W.M.); 3 Biochemical Institute for Environmental Carcinogens (BIU), Prof. Dr. Gernot Grimmer-Foundation, Lurup 4, 22927 Grosshansdorf, Germany; albrecht.seidel@biu-grimmer.de; 4 Institute of Pharmacy, Freie Universität Berlin, Königin-Luise-Strasse 2–4, 14195 Berlin, Germany

**Keywords:** *Brassica* vegetable, broccoli, DNA adducts, glucosinolates, neoglucobrassicin, *N*-methoxy-indole-3-carbinol, sulfotransferase

## Abstract

We previously found that feeding rats with broccoli or cauliflower leads to the formation of characteristic DNA adducts in the liver, intestine and various other tissues. We identified the critical substances in the plants as 1-methoxy-3-indolylmethyl (1-MIM) glucosinolate and its degradation product 1-MIM-OH. DNA adduct formation and the mutagenicity of 1-MIM-OH in cell models were drastically enhanced when human sulfotransferase (SULT) 1A1 was expressed. The aim of this study was to clarify the role of SULT1A1 in DNA adduct formation by 1-MIM-OH in mouse tissues in vivo. Furthermore, we compared the endogenous mouse Sult1a1 and transgenic human SULT1A1 in the activation of 1-MIM-OH using genetically modified mouse strains. We orally treated male wild-type (wt) and Sult1a1-knockout (ko) mice, as well as corresponding lines carrying the human *SULT1A1-SULT1A2* gene cluster (tg and ko-tg), with 1-MIM-OH. *N*^2^-(1-MIM)-dG and *N*^6^-(1-MIM)-dA adducts in DNA were analysed using isotope-dilution UPLC-MS/MS. In the liver, caecum and colon adducts were abundant in mice expressing mouse and/or human SULT1A1, but were drastically reduced in ko mice (1.2–10.6% of wt). In the kidney and small intestine, adduct levels were high in mice carrying human *SULT1A1-SULT1A2* genes, but low in wt and ko mice (1.8–6.3% of tg-ko). In bone marrow, adduct levels were very low, independently of the SULT1A1 status. In the stomach, they were high in all four lines. Thus, adduct formation was primarily controlled by SULT1A1 in five out of seven tissues studied, with a strong impact of differences in the tissue distribution of mouse and human SULT1A1. The behaviour of 1-MIM-OH in these models (levels and tissue distribution of DNA adducts; impact of SULTs) was similar to that of methyleugenol, classified as “probably carcinogenic to humans”. Thus, there is a need to test 1-MIM-OH for carcinogenicity in animal models and to study its adduct formation in humans consuming brassicaceous foodstuff.

## 1. Introduction

Glucosinolates are characteristic secondary metabolites of Brassicales, a plant order that comprises many food plants, e.g., cabbage, broccoli, turnip, mustard, capers, cress, radish and papaya. They are β-D-thioglucoside-*N*-sulphates containing a variable side chain [1,2,3,4]. The glucosinolates are used by the plant as a defence against microbial infections and herbivores [5,6,7,8,9]. This defence system additionally involves myrosinases, stored separately from the glucosinolates in the intact plant. After injury of the plant tissue, this glucosidase comes into contact with the glucosinolates, converting them to unstable aglycones, which rearrange to various biologically active products [10,11,12]. These products are able to damage other organisms, in particular infecting bacteria and fungi as well as herbivores. Overall, isothiocyanates are the most important active forms. They are electrophiles, and as such, they can chemically react with numerous cellular nucleophiles, including DNA, RNA and proteins. Thus, they will find target structures in any potential enemy, including humans consuming brassicaceous foods. In fact, serum albumin adducts of allyl isothiocyanate, benzyl isothiocyanate, phenylethyl isothiocyanate and sulforaphane–isothiocyanates derived from the glucosinolates sinigrin, glucatropaeolin, gluconasturtiin and glucoraphanin, respectively, were detected in human blood samples [13,14]. Likewise, we observed the formation of 1-methoxy-3-indolylmethyl (1-MIM) histidine adducts in serum albumin, haemoglobin and proteins from the internal tissues of mice orally treated with neoglucobrassicin (1-methoxy-3-indolylmethyl glucosinolate, 1-MIM GL) or its decomposition product 1-methoxy-3-indolylmethyl alcohol (1-MIM-OH, *N*-methoxy-indole-3-carbinol) [15]; these adducts were also formed in the serum albumin and haemoglobin of mice receiving a pak choi diet [16] and humans consuming broccoli [17]. Adduct formation with proteins and RNA may not be detrimental, and be tolerable, if the modification level remains low, as these macromolecules are present in many copies in a cell and are continuously renewed by degradation and transcription/translation. However, protein adducts represent biomarkers demonstrating that reactive metabolites of glucosinolates are present in blood or internal tissues. Positive adduct results in humans and animal models in vivo support the potential significance of results from in vitro models, e.g., genotoxicity.

Thus, juices of Brassicales were mutagenic in the Ames test, which uses *Salmonella typhimurium* as target cells [18,19]. Likewise, several purified glucosinolates and their breakdown products have demonstrated mutagenic and other genotoxic activities in in vitro tests with bacterial and mammalian target cells [18,20,21,22,23,24,25,26,27]. In some cases, mutagenicity was associated with the formation of DNA adducts. Taken together, these findings point to a possible carcinogenic risk of glucosinolates.

However, there is no evidence for such a risk from epidemiological studies. Quite the contrary, in numerous studies (usually retrospective case–control studies), high consumption of *Brassica* vegetables was negatively associated with the incidence of common cancer types (general reviews: [28,29,30,31]; meta-analyses for certain cancer types: [32,33,34]). Indeed, several molecular–biological effects that might counteract carcinogenesis in some situations have been observed in humans and experimental animals given *Brassica* vegetables, purified glucosinolates or their breakdown products. The most prominent effects are the activation of the transcription factors Nrf2 (nuclear factor erythroid-related factor 2) and AhR (arylhydrocarbon receptor). The activation of Nrf2 leads, amongst others, to the induction of enzymes (e.g., glutathione transferases) that mediate the detoxification of chemically reactive metabolites of xenobiotics and reduce oxidative stress [35,36,37,38,39,40]. Likewise, the activation of AhR leads to the induction of various xenobiotic-metabolizing enzymes (other than those regulated by Nrf2) [41,42], modulates cellular differentiation and immuno surveillance [43,44,45], and protects intestinal stem cells against genotoxic stress by stimulating DNA damage response (transient cell-cycle arrest, and DNA repair or elimination of damaged cells by apoptosis) [46]. These mechanisms adapt the organism to an increased burden of chemically reactive species, and they appear meaningful given the massive formation of reactive species from glucosinolates. Protection extends to reactive species formed from various chemicals other than glucosinolates, e.g., the mycotoxin aflatoxin B_1_ [47,48] and polycyclic aromatic hydrocarbons [49,50]. Thus, there may be a net benefit from exposure to glucosinolates in some situations.

However, more than 130 different glucosinolates have been detected in Brassicales [1,3]. They substantially differ in the profile of their biological activities. Hence, beneficial effects of some congeners may overshadow adverse effects of other congeners. Therefore, it is important to study and assess risks of individual congeners.

**Scheme 1:**
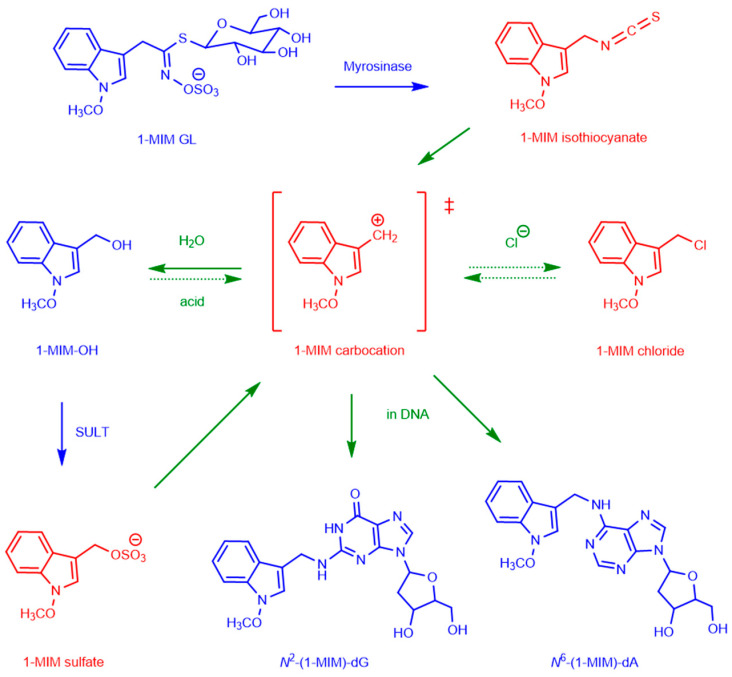
Activation pathway of 1-methoxy-3-indolylmethyl (1-MIM GL) with the structural formulas of the test compound, 1-methoxy-3-indolylmethyl alcohol (1-MIM-OH) and the DNA adducts studied. Substances marked in blue: chemically relatively stable under physiological conditions (and available as purified compounds); substances marked in red: short-lived, reactive species; blue arrows: enzyme-mediated reactions; green arrows: spontaneous reactions; dashed arrows: reactions that have been observed with other benzylic compounds, but not studied with metabolites of 1-MIM GL (discussed in the main text). SULT, sulfotransferase.

In an early study, we systematically investigated twelve purified glucosinolates on myrosinase-mediated DNA adduct formation in a cell-free system and on mutagenicity in *Salmonella typhimurium* strains [27]. The highest mutagenicity and DNA reactivity was found with 1-MIM GL. Later, we detected that expression of human SULT1A1 in bacterial and mammalian target cells leads to further enhancement of the mutagenicity of 1-MIM GL (and its DNA adduct formation) via the reactivation of its major degradation product, 1-MIM-OH [51]. The chemical structures of the DNA adducts were identified as *N*^2^-(1-MIM)-dG and *N*^6^-(1-MIM)-dA (structural formulas in Scheme 1). Subsequently, we demonstrated that feeding rats with broccoli or cauliflower [52,53], feeding mice a pak choi diet [16], and oral administration of 1-MIM-GL or 1-MIM-OH in mice [54] led to the formation of these DNA adducts [*N*^2^-(1-MIM)-dG and *N*^6^-(1-MIM)-dA] in the liver, gut and other tissues.

In conclusion, 1-MIM GL is a potent genotoxicant in in vitro systems and animal models. Two activation mechanisms, including specific enzymes that mediate the activation, have been identified in in vitro systems (Scheme 1): (i) 1-MIM GL is activated by plant myrosinase to become an ultimate mutagen, presumably 1-MIM isothiocyanate, identified as a short-lived intermediate in the myrosinase-mediated conversion of 1-MIM GL to 1-MIM-OH [55,56]. (ii) The second activation pathway starts from 1-MIM-OH and is mediated by human SULT1A1 in the presence of its co-substrate, 3′-phosphoadenosine-5′-phosphosulphate.

The SULT genetics vary substantially between human and mouse, in particular regarding the SULT1A subfamily [57]. Most mammalian species (except primates) have a single *SULT1A* gene, *SULT1A1*. Humans bear four *SULT1A* genes (*SULT1A1*, *SULT1A2*, *SULT1A3*, *SULT1A4*) as a result of gene duplications followed by mutations. *SULT1A3* and *SULT1A4* are separate genes, but the encoded protein (SULT1A3/4) is identical. The individual human SULT1A forms differ in their substrate specificities and regulation (e.g., tissue distribution) among themselves and compared to their mouse orthologue, Sult1a1 [57]. Thus, human SULT1A1, but not SULT1A2 and SULT1A3/4, expressed in *Salmonella typhimurium*, activated 1-MIM-OH to become a mutagen. Likewise, it is important to know that human SULT1A1 shows a much broader tissue distribution than mouse Sult1a1 (addressed in more detail in successive sections).

Based on the available data, 1-MIM GL is unique among the glucosinolates by forming high levels of DNA adducts in murine tissues. And it is special regarding its SULT-mediated activation, not observed with other glucosinolates (differing in the side chain). This accordance evoked the following questions: Is activation by SULTs the reason for the strong effect of 1-MIM GL? Is a specific SULT form, in particular SULT1A1/Sult1a1, important? Are there differences when either mouse Sult1a1 or human SULT1A1 is used for activation in an intact organism—in particular regarding the tissue distribution of the DNA adducts? Is high adduct formation restricted to tissues with expression of the activating enzyme or is there a transfer of the reactive intermediate between tissues? Previously, we constructed mouse lines with a disrupted *Sult1a1* gene (subsequently termed ko) [58] and/or a functional human *SULT1A1-SULT1A2* transgene (subsequently termed ko-tg and tg, respectively) [59]. These genetic manipulations had strong impacts on the level and tissue distribution of DNA adducts formed by various genotoxic carcinogens (compiled in the supplementary material). Here, we report findings on the DNA adduct formation by 1-MIM-OH in this model. The findings may be useful to estimate the relative sensitivity of mice and humans for adverse effects of 1-MIM GL, as well as to predict potential target sites.

## 2. Results

### 2.1. General Findings on DNA Adducts in Control and 1-MIM-OH-Treated Mice

**Figure 1:**
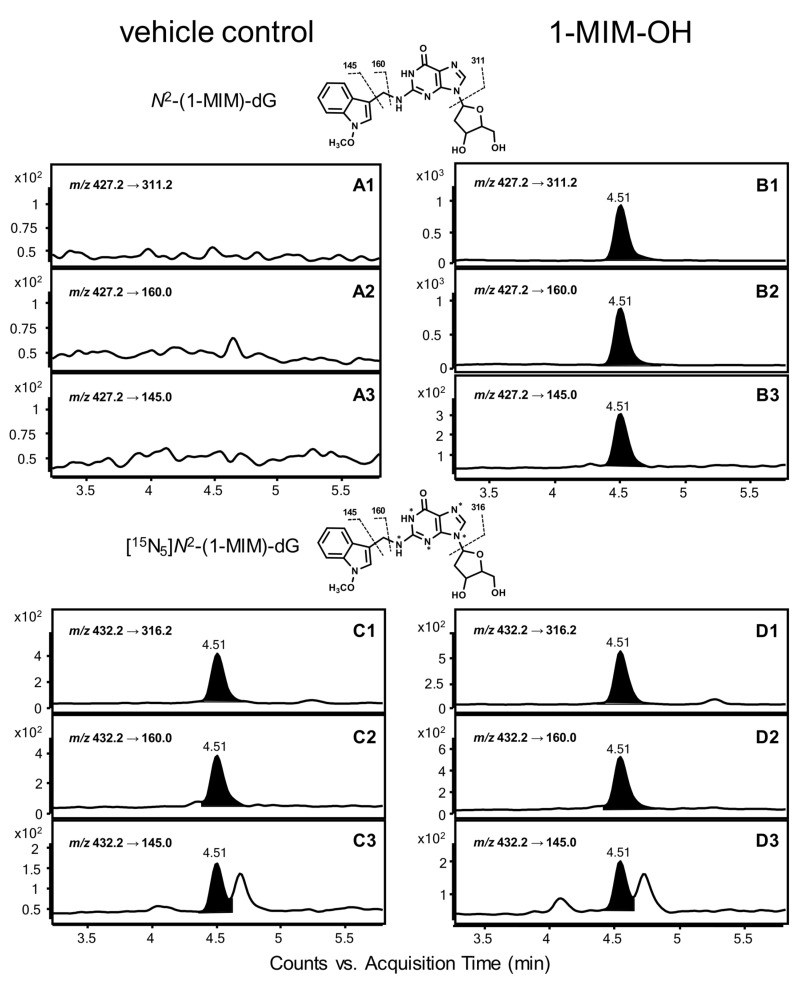
Representative primary data in the analysis of *N*^2^-(1-MIM)-dG adducts using isotope-dilution multiple reaction monitoring (MRM). The DNA was spiked with the isotopically labelled adduct standards, [^15^N_5_]*N*^2^-(1-MIM)-dG and [^15^N_5_]*N*^6^-(1-MIM)-dA, digested to 2’-deoxynucleosides (dN) and followed by separation by UPLC with MS/MS detection. The graphs show data for caecal samples from a vehicle-treated wt mouse (**A1**–**A3**,**C1**–**C3**) and a 1-MIM-OH-treated wt mouse (**B1**–**B3**,**D1**–**D3**). The transition *m/z* 427.2 → 311.2 (loss of deoxyribose) was used as a quantifier for the analyte (**A1**,**B1**), together with the corresponding signal (*m/z* 432.2 → 316.2) of the isotopically labelled internal standard (36.15 fmol on column) (**C1**,**D1**). The remaining panels show qualifier signals [60]. Signals attributed to the analyte or the internal standard are marked in black. The asterisks in the structural formula of the internal standard mark the position of the ^15^N label. The *N*^2^-(1-MIM)-dG adduct level in the 1-MIM-OH-treated mouse was calculated as 1406 per 10^8^ dN. No adducts were detected in the control animal (only treated with the vehicle tricaprylin). Note that the ordinate scale was adjusted to the signal sizes.

**Figure 2:**
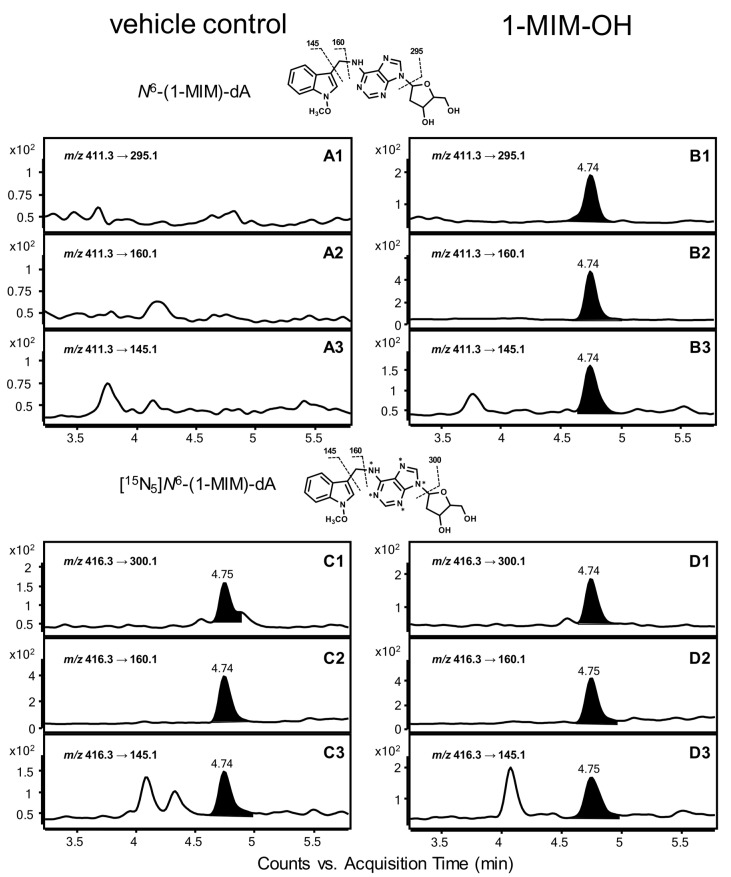
Representative primary data in the analysis of *N*^6^-(1-MIM)-dA adducts using isotope-dilution multiple reaction monitoring (MRM). The DNA was spiked with the isotopically labelled adduct standards, [^15^N_5_]*N*^2^-(1-MIM)-dG and [^15^N_5_]*N*^6^-(1-MIM)-dA, digested to 2’-deoxynucleosides (dN) and followed by separation by UPLC with MS/MS detection. Here, we show data for caecal samples from a vehicle-treated wt mouse (**A1**–**A3**,**C1**–**C3**) and a 1-MIM-OH-treated wt mouse (**B1**–**B3**,**D1**–**D3**). The transition *m*/*z* 411.3 → 160.1 (loss of dA) was used as quantifier for the analyte (**A2**,**B2**), together with the corresponding signal (*m/z* 416.3 → 160.1) of the isotopically labelled internal standard (59.85 fmol on column) (**C2**,**D2**). The remaining panels show qualifier signals [60]. Signals attributed to the analyte or the internal standard are marked in black. The asterisks in the structural formula of the internal standard mark the position of the ^15^N label. The *N*^6^-(1-MIM)-dA adduct level in the 1-MIM-OH-treated mouse was calculated as 1074 per 10^8^ dN. No adducts were detected in the control animal (only treated with the vehicle tricaprylin). Note that that the ordinate scale was adjusted to the signal sizes.

In this study, we determined the levels of *N*^2^-(1-MIM)-dG and *N*^6^-(1-MIM)-dA in DNA in mouse tissues using ultra-performance liquid chromatography (UPLC) coupled with mass spectrometry. The mass spectrometry involved isotope-dilution multiple reaction monitoring (MRM), using three mass/charge (*m*/*z*) transitions for each analyte. Exemplary chromatographic traces for *N*^2^-(1-MIM)-dG and *N*^6^-(1-MIM)-dA adducts are shown in Figure 1 and Figure 2, respectively. The usage of three *m/z* transitions guaranteed high specificity of the method. The use of isotopically labelled internal standards allowed for accurate quantification. The limit of detection (LOD) of the method is 7 and 10 adducts per 10^8^ dN, respectively [60].

A total of 28 tissue samples from untreated mice (one from each tissue and mouse line) were investigated on the presence of *N*^2^-(1-MIM)-dG and *N*^6^-(1-MIM)-dA in DNA. All results were negative. On the contrary, *N*^2^-(1-MIM)-dG was detected in 134 out of 141 tissue samples from 1-MIM-OH-treated mice (details in Table 1). Six out of the seven negative results were obtained with bone marrow. In the 15 remaining bone marrow samples, *N*^2^-(1-MIM)-dG adducts were detected, but their levels were very low (≤15 adducts per 10^8^ dN). The level of the second adduct investigated, *N*^6^-(1-MIM)-dA, was somewhat lower than that of the dG adduct in most samples, except the stomach, where the levels of both adducts were nearly equal. *N*^6^-(1-MIM)-dA adducts were detected in 125 out of the 141 tissue samples from 1-MIM-OH-treated mice (details in Table 2). Again, the majority of the negative results (13 out of 16) were obtained in the bone marrow. Apart from the somewhat lower sensitivity (due to lower abundance and a higher LOD), the findings with *N*^6^-(1-MIM)-dA matched those with *N*^2^-(1-MIM)-dG.

The levels of *N*^2^-(1-MIM)-dG and *N*^6^-(1-MIM)-dA adducts exceeded the LOD more than 100-fold in 67 and 36 individual samples, respectively. The lack of any positive adduct results in untreated mice, the abundance of adducts in 1-MIM-OH-treated mice and the very strong signals in many samples substantiate the specificity and sensitivity of the detection method used.

### 2.2. Adducts in wt Mice

This part of the study is a repeat of an experiment conducted previously [54], except that bone marrow rather than lung was used in the present study, the other six tissues being equal in both studies. The levels of *N*^2^-(1-MIM)-dG (Table 1) and *N*^6^-(1-MIM)-dA (Table 2) adducts in the various tissues were similar in both studies (first two result columns in the tables). Consequently, the tissue-dependent variation in the adduct levels was similar in both studies. In wt animals, adduct levels were high in the liver, large intestine and stomach, and low in the remaining tissues.

### 2.3. Impact of Sult1a1 Knockout on Adduct Formation

*Sult1a1* knockout led to a collapse of the formation of 1-MIM DNA adducts in three out of the four tissues that showed high adduct levels in the wt: the levels in ko mice, compared to the wt, amounted to 2.2 and 2.3% for *N*^2^-(1-MIM)-dG and *N*^6^-(1-MIM)-dA adducts in liver; the corresponding values for caecum were 1.1 and 1.2%, and for colon, 4.6 and 10.6%, respectively (second and third result columns in Table 1 and Table 2).

In the stomach, the fourth tissue with high adduct levels in wt mice, the reduction was much smaller, namely to 59% (statistically not significant, *p*~0.06) and 53% (*p* < 0.01) of the wt level for the dG and dA adducts, respectively. As a consequence of this moderate reduction, it became the tissue with highest adduct levels in ko mice, the levels being approximately 15 times higher than in the liver, the tissue with the second highest adduct level.

The remaining tissues investigated (small intestine, kidney and bone marrow) showed low adduct levels in both genotypes, wt and ko. In small intestine, there were no statistical differences in the adduct levels between these mouse lines. In bone marrow, adduct levels in wt and ko mice (as well as the other genotypes) were similar and near to the LOD. In kidney, adduct levels were statistically significantly lower in ko mice than the (already low) levels in the wt.

### 2.4. Impact of the Human SULT1A1-SULT1A2 Transgene on Adduct Formation

A potential activation of a compound by transgenic enzymes is best seen in animal models with low endogenous activation, i.e., if the transgene is expressed in *Sult1a1*-ko mice in the case of 1-MIM-OH. The ratios of the adducts of ko-tg (as well as wt and tg) and ko animals in the various tissues, calculated from the data presented in Table 1 and Table 2, are shown in Table 3. It is obvious that human SULT1A1 (and/or SULT1A2) strongly enhanced the DNA adduct formation (up to 100-fold) in ko-tg as compared to ko mice in tissues with high SULT1A1/2 expression (liver, small intestine, caecum, colon and kidney). This result is particularly interesting for the small intestine and kidney, as these tissues only manifested very low adduct levels when wt mice were utilised.

## 3. Discussion

### 3.1. Tissue Distribution of DNA Adduct Formation and SULT1A Expression

Adduct formation was high in the liver, caecum and colon of wt mice, but collapsed in ko mice (to 1.1–10.6% of the wt levels). These three tissues are characterized by high expression of *Sult1a1* on mRNA and protein levels in wt mice (see Section 1). This finding indicates that Sult1a1 was the predominant activator of 1-MIM-OH in these tissues. Likewise, 1-MIM-OH induced high levels of adducts in these tissues in humanised mice (ko-tg), expressing human SULT1A1/2 instead of mouse Sult1a1.

A fourth tissue, the stomach, demonstrated high adduct levels, but with a low impact of mouse Sult1a1 and human SULT1A1/2, implying that other activation mechanisms were important. It is interesting to know that the expression of *Sult1a1* is very low in the stomach of wt mice [61]. However, the expression of *Sult1c2* is higher in the stomach than in any other tissue investigated, and several other *Sult* forms (*1b1*, *1c1*, *2b1*, *1d1* and 5a1) are expressed in this tissue at appreciable levels [61]. It is not known whether any of these Sult forms are able to activate 1-MIM-OH. However, we found that human SULT1C2 (using the original nomenclature), expressed in Ames’s *Salmonella typhimurium* strain TA100, efficiently activates 1-MIM-OH to become a mutagen [52] (and Table S1 in Supplementary Material). Chemical activation in the acidic milieu of the stomach is another possibility: protonation of the hydroxyl group of 1-MIM-OH could lead to the elimination of water with subsequent formation of the 1-MIM carbocation (Scheme 1). The protonated 1-MIM-OH and the 1-MIM carbocation are charged molecules, which argues against absorption into gastric cells. Moreover, the cation is extremely short-lived, and the protonated 1-MIM-OH would be deprotonated immediately in the nearly neutral intracellular milieu. However, the 1-MIM carbocation could react with chloride anions (or other nucleophiles) in the stomach lumen, creating a reactive but uncharged reactive species (e.g., 1-MIM chloride), which could be transferred into cells to form the adducts. Exchange of the leaving group has been observed with many compounds reactive at benzylic positions [62].

In two further tissues—the small intestine and kidney—adduct levels were low in wt and ko mice, but drastically increased in tg and ko-tg mice (up to 27- and 57-fold, compared to wt and ko mice, respectively—Table 3). This difference in adduct formation correlates with the fact that transgenic human *SULT1A1-SULT1A2* is highly expressed in these tissues, whereas the endogenous *Sult1a1* is not expressed (or weakly expressed) (references in the legend to Table 3). We conclude that human SULT1A1/2 is the principal activator of 1-MIM-OH in the kidney and small intestine of tg and ko-tg mice

In the bone marrow, adduct formation was very low and independent of the SULT1A status. Implications of these findings for the genotoxicity testing are addressed in Section 3.2.

As indicated in Table 3, disruption of Sult1a1 led to drastic decreases in the adduct formation in the liver (by a factor of 44, mean of values for *N*^2^-(1-MIM)-dG and *N*^6^-(1-MIM)-dA), caecum (factor 87) and colon (factor 15.5), but had no impact or a much weaker impact on the adducts in the stomach (factor 1.8), small intestine (factor 1.0), kidney (factor 3.4) and bone marrow (factor 0.8). This finding argues for local activation rather than activation in the liver (or large intestine) followed by transfer of the reactive intermediate, 1-MIM sulphate, to other tissues as a mechanism underlying the adduct formation. This view is supported by the drastic increases in the adduct levels in the small intestine and kidney in mouse lines carrying the human *SULT1A1-SULT1A2* transgene (which is strongly expressed in these tissues unlike the endogenous *Sult1a1*).

### 3.2. Low Adduct Formation in Bone Marrow—Impact on Genotoxicity Testing

Bone marrow was the tissue with the lowest level of adduct formation by 1-MIM-OH. In fact, the adduct level was below the LOD in some animals and up to 2-fold that of the LOD in other animals, apparently without any impact of the SULT1A1 status. This finding is important, as the bone marrow micronucleus test (usually conducted in mice) is the most widely used in vivo mutagenicity test [63,64]. For many drugs and industrial chemicals, it is the only in vivo mutagenicity test conducted. Other tests involve the analysis of chromosomal aberrations in bone marrow cells or of micronuclei in peripheral erythrocytes (originating from bone marrow). We would expect 1-MIM-OH to be negative in such tests, using the DNA adducts as a biomarker of exposure (in this case, very low) to the reactive intermediates. Similar situations may exist for other mutagens requiring activation by SULT1A enzymes.

### 3.3. Comparison of 1-MIM-OH with Methyleugenol and other Compounds Studied for DNA Adduct Formation in Mouse Lines with Modified SULT1A1 Status

SULTs are involved in the formation of electrophilically reactive metabolites from many compounds. We expressed the various human SULTs in Ames’s *Salmonella* strains, the most popular mutagenicity test system. We found approximately 100 compounds that were inactive in conventional strains but mutagenic in strains expressing an appropriate human SULT, or they demonstrated clearly enhanced mutagenicity in a SULT-expressing strain ([65] and later publications from our laboratory). The largest number of compounds (>50) was activated by SULT1A1 among the different human forms (relevant data in Tables S1 and S2 in the Supplementary Material). Moreover, SULT1A1 is the most abundant SULT form in the human regarding tissue distribution and protein levels [66,67]. In previous studies, we investigated eleven other compounds for DNA adduct formation or other genotoxic effects in mouse models with genetically modified SULT1A1 status. The impact of Sult1a1 knockout or transgenic SULT1A/2 varied substantially among these compounds (Table S3 in the Supplementary Material). Dissimilarities may be owed to the bioactivation of some test compounds in some murine tissues by SULT1A-independent mechanisms (e.g., other Sult forms); the distribution of some reactive sulpho conjugates, but not others, via circulation; and different substrate specificities of mouse and human SULT1A enzymes. All of these mechanisms were verified in some examples. Overall, the adduct formation by 1-MIM-OH in mice with modified SULT1A1 status showed the greatest similarity with that seen with methyleugenol (key results presented in Table 4).

Similarities were observed for the following parameters:

- Methyleugenol and 1-MIM-OH formed DNA adducts at the same sites, the exocyclic amino groups of adenine and guanine.
- The adduct levels per dose unit were in same range; e.g., the combined dA and dG adducts in wt livers were 49 per 10^8^ dN per mg test compound with 1-MIM-OH (calculated from data in Table 1 and Table 2, means from previous and actual study); the corresponding value for methyleugenol was 31 per 10^8^ dN per mg test compound (data for dG adducts from Table 4 and, for dA adducts, from [68]).
- In wt animals, high adduct levels were formed in the liver, caecum and stomach. On the contrary, adducts were not detected (methyleugenol) or were low (1-MIM-OH) in the kidneys of wt mice.
- The knockout of Sult1a1 drastically reduced DNA adduct formation in the liver and caecum (to ≤3.1% of the wt level); on the contrary, adduct levels in the stomach were unaffected (methyleugenol) or only slightly reduced (1-MIM-OH) in ko mice, indicating an SULT1A1-independent activation mechanism. Thus, the stomach was clearly the tissue with the highest adduct levels induced with both compounds in ko mice.
- SULT1A1 humanization drastically enhanced DNA adduct formation in the kidney (ko-tg versus wt) by 13.1–17.6-fold (1-MIM-OH, Table 1 and Table 2) and ≥37-fold (methyleugenol, Table 4).

Methyleugenol is classified by the International Agency for Research on Cancer (IARC), Lyon, as “probably carcinogenic to humans” (Group 2A). This classification was “based on ’sufficient’ evidence for cancer in experimental animals and ’strong’ mechanistic evidence, including studies in humanised mice and supported by mechanistic studies in exposed humans” [69]. Up to date, it is the only food-borne phytochemical in group 2A (or 1).

Methyleugenol has demonstrated carcinogenic activity in long-term studies in rats and mice. The liver was the major target organ, but weaker carcinogenic effects were detected in many other tissues [70,71]. 1-MIM-OH has not been tested for carcinogenicity in experimental animals. The results of our study suggest that wt mice may be a suitable model for such studies. We could predict the detection of hepatocarcinogenicity for 1-MIM-OH in this model, taking into account that it forms hepatic DNA adducts at levels (per dose unit) similar to those observed with methyleugenol in wt mice. In fact, a good quantitative correlation has been observed between DNA adduct formation and carcinogenesis, induced by diverse genotoxicants in the liver of mice (and rats) [72]. In other experiments, in which tg mice were treated sub-chronically (for 90 d) with 1-MIM-OH, hepatic adduct levels accumulated over time and 1-MIM-OH induced a gene expression profile similar to the expression signature caused by known genotoxic hepatocarcinogens [73].

The use of tg or ko-tg mice in carcinogenicity studies would enhance the relevance of the findings for humans and increase the potential for the detection of extrahepatic effects.

The detection of methyleugenol DNA adducts in numerous human tissue specimens [74,75] was an import reason for classifying methyleugenol as “probably carcinogenic to humans” (Group 2A). 1-MIM-OH DNA adducts have not yet been studied thoroughly in human tissues. However, methyleugenol and 1-MIM-OH adducts were investigated concurrently in ten human lung samples [75]. 1-MIM-OH adducts were not detected in any sample, whereas methyleugenol adducts were present in all samples, with similar LODs for both adduct types. Verified data on the presence of 1-MIM-OH DNA adducts in other human tissues are missing, apart from a positive result in buccal mucosa in a subject who chewed a pound of raw broccoli [52]. However, our laboratory has devised a method for the detection of 1-MIM haemoglobin adducts in mice [15]. Using a modification of this method that enhances its sensitivity, we detected 1-MIM haemoglobin adducts in many human blood samples (without deliberate exposure to 1-MIM GL via the diet) [17]. These adducts can be formed from 1-MIM GL, a secondary plant metabolite, after breakdown to an electrophilically isothiocyanate or sulphation of 1-MIM-OH, another breakdown product of 1-MIM GL (as shown in Figure 1). We do not know of any other exposure in humans that could lead to the formation of these adducts. Therefore, the haemoglobin adducts detected demonstrate that humans are exposed to reactive metabolites of 1-MIM GL/1-MIM-OH.

## 4. Materials and Methods

### 4.1. Test Material and Adduct Standards

We used 1-MIM-OH, rather than 1-MIM GL, as the test compound for two reasons: (i) The activation of 1-MIM GL requires an additional enzyme, a myrosinase-like glucosidase, and, as shown in the in vitro models, 1-MIM GL may be activated into two reactive metabolites able to form 1-MIM DNA adducts; this higher complexity would have complicated the interpretation of the results. (ii) Even though a chemical synthesis of 1-MIM GL has been described [76], it involves many steps and yields only moderate quantities—practically, 1-MIM GL has to be purified from plant material in tedious procedures, a strong limitation for the size of animal experiments and the dosage levels.

1-MIM-OH was synthesized as reported previously [51]. It was stored at −80 °C. Its purity, checked by HPLC-UV on the day of usage, was >99%. [^15^N_5_]*N*^2^-(1-MIM)-dG and [^15^N_5_]*N*^6^-(1-MIM)-dA were prepared as described previously [60].

### 4.2. Mouse Lines

Wild-type FVB/N mice (subsequently termed wt mice) were purchased from Harlan (Borchen, Germany). The generation of FVB/N mice transgenic for the human *SULT1A1-SULT1A2* gene cluster is described elsewhere [59]. The line termed tg1 in the original study was used; it contains multiple copies of the human *SULT1A1-SULT1A2* gene cluster integrated into chromosome 9. For the maintenance of the line, homozygous animals (tg1-homo) were used. The construction of *Sult1a1* knockout mice in the FVB/N background was described in a previous study [58]. The transgenic line tg1 was bred with ko mice to generate a line homozygous for both traits (termed ko-tg1-homo). For the present study, hemizygous transgenic animals were used, whereas the knockout was homozygous. They were obtained as an F1 progeny of tg1-homo mice bred with wt mice, resulting in mice subsequently termed tg. Analogously, an F1 progeny from ko and ko-tg1-homo was created (subsequently termed ko-tg).

Global hepatic gene expression was compared in wt, tg and ko mice (5 male animals per group, at the age of 8 weeks) using Mouse Genome 430 2.0 Arrays (covering 39,000 transcripts; Affymetrix, Santa Clara, CA, USA) according to the manufacturer’s recommendations. The genetic manipulations did not lead to altered expression of any genes encoding other SULT forms or other xenobiotic-metabolizing enzymes. Among the remaining genes, only a handful genes showed changes in expression exceeding a factor of 1.5 in tg or ko mice.

### 4.3. Treatment of Animals

In a previous study, male wt mice were treated with 1-MIM-OH by gavage, varying the dose level and the time period between treatment and killing [54]. A single dose of 106 mg (600 µmol) per kg of body mass produced high levels of DNA adducts and was well tolerated. Maximal adduct levels were observed 8 h after treatment. Therefore, these conditions were used in the present study.

We wanted to treat all animals from the different lines concurrently with 1-MIM-OH or the vehicle only (tricaprylin, 1.7 mL/kg of body mass), six animals per group. Although we did not detect any *N*^2^-(1-MIM)-dG or *N*^6^-(1-MIM)-dA in any control animal in the previous studies [16,54], an equal number of vehicle control animals was used, because we studied additional endpoints not reported here. The animals had to be bred specifically for the experiment. Unfortunately, the production of male tg-ko mice was so low that the size of these groups had to be reduced to three. In addition, three tissue samples from a tg mouse were not available, as used for other investigations.

The mice were housed in individually ventilated cages under specific pathogen-free conditions with a 12 h dark/12 h light cycle. They received a glucosinolate-free semisynthetic diet (C1000, Altromin, Lage, Germany). At treatment, the animals were 8–10 weeks old.

The liver, small intestine and caecum were homogenised in a mortar at −196 °C (in liquid nitrogen). The other tissues were homogenised using a T10 basic Ultra-Turrax (IKA-Werke, Staufen, Germany). The homogenates were stored at −80 °C.

### 4.4. Analysis of DNA Adducts

DNA was isolated from tissue homogenates using Plasmid Midi Kits (Qiagen, Venlo, The Netherlands) according the manufacturer’s instruction. Samples of 25 µg of DNA were spiked with [^15^N_5_]*N*^2^-(1-MIM)-dG and [^15^N_5_]*N*^6^-(1-MIM)-dA as internal standards and hydrolysed to 2′-deoxynucleosides (dN) with micrococcal nuclease, phosphodiesterase type II and alkaline phosphatase according to our optimized protocol [54]. The hydrolysate was subjected to C_18_ solid-phase extraction [60] before the actual adduct analysis.

Sample separation and analyte detection were carried out with an Acquity UPLC system equipped with an Acquity UPLC BEH Phenyl column (1.7 µm, 2.1 × 100 mm) and a Xevo TQ triple quadrupole mass spectrometer (all from Waters, Eschborn, Germany). The analytes were ionized within an electrospray source operating in the positive ion mode (ESI+). The 2′-deoxynucleoside adducts were quantified by isotope-dilution multiple reaction monitoring (MRM) using the following mass transitions (asterisks refer to the internal standard): *m*/*z* 411.3 (416.3*) → 160.1 (loss of dA) for *N*^6^-(1-MIM)-dA and *m*/*z* 427.2 (432.2*) → 311.2 (316.2*) (loss of deoxyribose) for *N*^2^-(1-MIM)-dG. For each adduct, two further transitions were used as qualifiers. Further details, including chromatographic conditions and settings of the MS/MS detector, have been published elsewhere [60].

## 5. Conclusions

In this study, we demonstrate that SULTs are important in the activation of 1-MIM-OH in the mouse and that a specific form, Sult1a1, determines the adduct formation in many tissues. By replacing Sult1a1 by human SULT1A1/2 (in ko-tg mice), we showed that human SULT1A enzymes are also able to efficiently activate 1-MIM-OH in an in vivo model. In fact, adduct levels were strongly increased (up to 23-fold) in the small intestine and kidneys of ko-tg compared to wt mice, which can be explained by the wider tissue distribution of the human enzyme. Our findings indicate that 1-MIM sulphate primarily forms DNA adducts at the site of its formation, rather than being transferred between tissues. Adduct levels formed by 1-MIM-OH, their tissue distribution and the impact of the SULT1A1 status of the mouse lines used were similar to those previously detected with methyleugenol, which was classified by the IARC as “probably carcinogenic to humans” (Group 2A). For both compounds, macromolecular adducts (methyleugenol DNA adducts in liver and lung; 1-MIM protein adducts in serum albumin adducts in blood) indicate that many human subjects are exposed to these pro-genotoxicants and their activated intermediates. Thus, there is a need to test 1-MIM-OH for carcinogenicity in animal models, ideally models expressing human SULT1A1/2, to pinpoint target tissues, and to clarify whether the actual exposure results in a relevant carcinogenic risk.

## Figures and Tables

**Table 1:** Influence of the SULT1A1 status on the levels and tissue distribution of *N^2^*-(1-MIM)-dG adducts in mice treated with 1-MIM-OH ^a^.

Tissue	*N*^2^-(1-MIM)-dG per 10^8^ dN, Mean ± SD of n Animals; % *of wt*				
	Previous Study [54]	Actual Study			
	wt	wt	ko	tg	ko-tg
Liver	4160 ± 1050 (5)	3010 ± 940 (6) *100*	66 ± 15 (6) *** *2.2*	6050 ± 1170 (6) *** *201*	6330 ± 3620 (3) ^ns^ *210*
Stomach	1450 ± 540 (5)	1510 ± 20 (6) *100*	890 ± 170 (6) ^ns^ *59*	1830 ± 310 (5) ^ns^ *121*	1710 ± 280 (2) ^ns^ *113*
Small intestine	74 ± 38 (5)	45 ± 25 (6) *100*	65 ± 15 (6) ^ns^ *146*	860 ± 310 (5) *** *1920*	1130 ± 460 (3) *** *2310*
Caecum	5730 ± 2950 (5)	2460 ± 1330 (6) *100*	28 ± 17 (6) ** *1.1*	1950 ± 1450 (5) ^ns^ *79*	850 ± 700 (3) ^ns^ *35*
Colon	1020 ± 490 (5)	820 ± 420 (6) *100*	37 ± 41 (6) ** *4.6*	1470 ± 1750 (6) ^ns^ *179*	340 ± 210 (3) ^ns^ *42*
Kidney	70 ± 29 (5)	108 ± 41 (6) *100*	32 ± 21 (5 + 1) ^b,^** *30*	1060 ± 450 (6) *** *983*	1760 ± 1250 (3) * *1620*
Bone marrow	n.t. ^c^	8 ± 4 (4 + 2) ^b^ *100*	5 ± 3 (2 + 4) ^b^ *68*	11 ± 3 (6 + 0) ^b^ *135*	12 ± 5 (3 + 0) ^b^ *149*
Lung	19 ± 13 (5)	n.t.	n.t.	n.t.	n.t.

^a^ Male mice (FVB/N, wild-type and genetically modified derivatives, 8–10 weeks old) received a single oral dose of 1-MIM-OH (600 µmol per kg body mass; 600 µmol = 106 mg) by gavage, using tricaprylin as the vehicle (wt, wild-type; ko, knockout of Sult1a1; tg, transgenic for human *SULT1A1-1A2* with intact endogenous *Sult1a1*; ko-tg, transgenic for human *SULT1A1-1A2* with disrupted endogenous *Sult1a1* gene). Additional animals (negative controls) only received the tricaprylin. Animals were killed 8 h after the treatment. The treatment scheme was equal in the previous study [54] and in the actual study, except that we used total small intestine in the actual study, whereas the jejunum was utilised in the initial study. Adducts were analysed using UPLC-MS/MS with an isotopically labelled internal standard. No adducts were detected in any tissue of any negative control animals, with a limit of detection (LOD) of 7 *N*^2^-(1-MIM)-dG per 10^8^ dN. Adduct levels in genetically modified mouse lines were statistically compared with those in the wild-type (of the actual experiment) using Student’s *t*-test: ns, not significant (*p* ≥ 0.5); *, 0.05 > *p* ≥ 0.01; **, 0.01 > *p* ≥ 0.001; *** 0.001 > *p*. ^b^ Adduct levels were below the LOD in some samples. In these cases, the number of animals studied was split in those with adduct levels above and below the LOD (first and second number in the parentheses). If adduct levels were below the LOD, they were set as ½ LOD for the calculation of the mean and SD. No statistical analysis was performed with bone marrow, since all of its adduct levels were very low, close to the LOD. ^c^ n.t., not tested in that study.

**Table 2:** Influence of the SULT1A1 status on the levels and the tissue distribution of *N^6^*-(1-MIM)-dA adducts in mice treated with 1-MIM-OH ^a^.

Tissue	*N*^6^-(1-MIM)-dA per 10^8^ dN, Mean ± SD of n Animals; *% of wt*				
	Previous Study [54]	Actual Study			
	wt	wt	ko	tg	ko-tg
Liver	1510 ± 510 (5)	1700 ± 500 (6) *100*	40 ± 15 (6) *** *2.3*	3910 ± 840 (6) *** *230*	3970 ± 2090 (3) * *234*
Stomach	1250 ± 640 (5)	1260 ± 400 (6) *100*	660 ± 190 (6) ** *53*	1800 ± 550 (6) ^ns^ *143*	1550 ± 490 (2) ^ns^ *123*
Small intestine	54 ± 40 (4 + 1) ^b^	39 ± 16 (6) *100*	30 ± 9 (6) ^ns^ *79*	500 ± 180 (5) *** *1300*	670 ± 310 (3) *** *1740*
Caecum	1640 ± 690 (5)	1650 ± 1040 (6) *100*	19 ± 17 (4 + 2) ^b,^** *1.2*	1250 ± 1980 (5) ^ns^ *100*	510 ± 390 (3) ^ns^ *100*
Colon	230 ± 90 (5)	350 ± 160 (6) *100*	37 ± 43 (6) *** *10.6*	600 ± 490 (6) ^ns^ *76*	103 ± 53 (3) * *31*
Kidney	22 ± 16 (3 + 2) ^b^	84 ± 29 (6) *100*	24 ± 12 (5 + 1) ^b,^*** *29*	580 ± 220 (6) *** *690*	1120 ± 880 (3) * *1340*
Bone marrow	n.t. ^c^	8 ± 4 (2 + 4) ^b^ *100*	8 ± 7 (1 + 5) ^b^ *102*	6 ± 2 (1 + 5) ^b^ *76*	7 ± 4 (1 + 2) ^b^ *93*
Lung	<LOD (0 + 5)	n.t.	n.t.	n.t.	n.t.

^a^ Male mice (FVB/N, wild-type and genetically modified derivatives, 8–10 weeks old) received a single oral dose of 1-MIM-OH (600 µmol per kg body mass; 600 µmol = 106 mg) by gavage, using tricaprylin as the vehicle (wt, wild-type; ko, knockout of Sult1a1; tg, transgenic for human *SULT1A1-1A2* with intact endogenous *Sult1a1*; ko-tg, transgenic for human *SULT1A1-1A2* with disrupted endogenous *Sult1a1* gene). Additional animals (negative controls) only received the tricaprylin. Animals were killed 8 h after the treatment. The treatment scheme was equal in the previous study [54] and in the actual study, except that we used total small intestine in the actual study, whereas the jejunum was utilised in the initial study. Adducts were analysed using UPLC-MS/MS with an isotopically labelled internal standard. No adducts were detected in any tissue of any negative control animals, with a limit of detection (LOD) of 10 *N*^6^-(1-MIM)-dA per 10^8^ dN. Adduct levels in genetically modified mouse lines were statistically compared with those in the wild-type (of the actual experiment) using Student’s *t*-test: ns, not significant (*p* ≥ 0.5); *, 0.05 > *p* ≥ 0.01; **, 0.01 > *p* ≥ 0.001; *** 0.001 > *p*. ^b^ Adduct levels were below the LOD in some samples. In these cases, the number of animals studied was split in those with adduct levels above and below the LOD (first and second number in the parentheses). If adduct levels were below the LOD, they were set as ½ LOD for the calculation of the mean and SD. No statistical analysis was performed with bone marrow, since all of its adduct levels were very low, close to the LOD. ^c^ n.t., not tested in that study.

**Table 3:** Fold increase in the levels of 1-MIM DNA adducts in various tissues of SULT1A1-expressing mice compared to Sult1a1-ko mice, and levels of expression of mouse and human *SULT1A1*.

Tissue	Adduct Ratio in SULT1A-Expressing versus Ko Mice [*N*^2^-(1-MIM)-dG; *N*^6^-(1-MIM)-dA] ^a^			Sult1a1 mRNA in wt Mice ^b^	Human SULT1A1 Protein in tg Mice ^c^
	wt	ko-tg	tg		
Liver	45; 43	96; 100	91; 99	++	++++
Stomach	1.7; 1.9	1.9; 2.3	2.1; 2.7	±	+
Small intestine	0.7; 1.3	16; 22	13; 17	±	++/+++
Caecum	88; 86	30; 27	69; 65	+++	+++
Colon	22; 9	9; 3	39; 16	++	+++
Kidney	3.3; 3.5	54; 47	33; 24	±	+++
Bone marrow	1.0; 0.6	1.2; 2.0	0.8; 1.4	n.s.	n.s.

^a^ Calculated from the data presented in Table 1 and Table 2. ^b^ From [61]. In this study, the mRNA expression of 11 Sult forms was studied in 14 tissues of male and female C57BL/6 mice, using the branched DNA signal amplification assay. Data in our table refer to approximately 8-week-old males. Data were presented in [61] as a bar plot. ±, close to the zero line of the figure (zero or minimally above zero); + to ++++, clear expression (the number of plus signs indicating the expression level; n.s., not studied. ^c^ From [59]. SULT1A1 and SULT1A2 proteins were detected by immunoblotting using antibodies that specifically recognize human SULT1A1 and SULT1A2, but not mouse Sult1a1. Expression of SULT1A2 was lower by a factor of 3–6 than that of SULT1A1. Expression of SULT1A1 in transgenic animals varied in different sections of the small intestine. It was higher in the duodenum and jejunum (+++) than in the ileum (++).

**Table 4:** Impact of SULT1A1 status on the DNA adduct formation by methyleugenol in mice ^a^.

Tissue	*N*^2^-(1-MIE)-dG per 10^8^ dN, Mean ± SD of 4 Animals; *% of wt*			
	wt	ko	tg	ko-tg
Liver	735 ± 342 *100*	23 ± 2 *3.1*	4500 ± 180 *612*	3770 ± 320 *513*
Stomach	520 ± 350 *100*	540 ± 190 *104*	580 ± 120 *112*	550 ± 90 *106*
Caecum	1210 ± 740 *100*	<LOD *<2.5*	2780 ± 330 *230*	1170 ± 310 *97*
Kidney	<LOD	<LOD	950 ± 70 *>3200*	1110 ± 320 *>3700*

^a^ Data from [68]. The animal model was the same as that used in this study (8-week-old male mice, 4 per group). Animals were treated with a single dose of methyleugenol (50 mg per kg body mass, gavage; 50 mg = 281 µmol) 6 h before sacrifice. The limit of detection (LOD) was 30 adducts per 10^8^ dN. It was used for calculating percent values in the corresponding cases. (LOD was lower for some other analyses, including that of liver from ko animals, due to the use of higher amounts of DNA). *N*^2^-(1-MIE)-dG, *N*^2^-(*trans*-methylisoeugenol-3′-yl)-2′-deoxyguanosine.

## Data Availability

Data is contained within the article and Supplementary Materials.

## Supplementary Materials

The following supporting information can be downloaded at: https://www.mdpi.com/article/10.3390/ijms25073824/s1. References [77,78,79,80,81,82,83,84,85,86,87,88,89,90,91,92,93,94,95,96,97,98,99,100] are cited in this Supplementary Material.

## Abbreviations

AhR, arylhydrocarbon receptor; BfR, Federal Institute of Risk Assessment; BIU, Biochemical Institute for Environmental Carcinogens; DIfE, German Institute of Human Nutrition; dN, 2-deoxynucleoside; IARC, International Agency for Research on Cancer; LOD, limit of detection; ko, knockout of *Sult1a1*; 1-MIM, 1-methoxy-3-indolylmethyl; 1-MIM GL, 1-methoxy-3-indolylmethyl glucosinolate; MRM, multiple reaction monitoring; MS, mass spectrometry; *m*/*z*, mass-to-charge ratio; Nrf2, nuclear factor erythroid-related factor 2; SULT, sulfotransferase (human or generic), in italics for genes, in romans for proteins and RNAs; Sult, sulfotransferase (mouse forms), in italics for genes, in romans for proteins and RNAs; SULT1A1/2, human SULT1A1 and/or SULT1A2 enzyme; tg, transgenic for the human *SULT1A1-SULT1A2* gene cluster; UPLC, ultra-performance liquid chromatography; wt, wild-type.
