# Supplementary material for: Formation of DNA Adducts by 1-Methoxy-3-indolylmethylalcohol, a Breakdown Product of a Glucosinolate, in the Mouse: Impact of the SULT1A1 Status—Wild-Type, Knockout or Humanised
Source: Int J Mol Sci. 2024 Mar 29;25(7):3824. doi: 10.3390/ijms25073824 (PMC11012018; doi:10.3390/ijms25073824)
Supplement: Supplementary file 1 [file ijms-25-03824-s001.zip › ijms-2813541-supplementary.pdf]

Formation of DNA Adducts by 1-Methoxy-3-Indolylmethylalcohol, a Breakdown Product of a Glucosinolate, in the Mouse: Impact of the SULT1A1 Status—Wild-Type, Knockout or Humanised

Supplementary material:

Formation of DNA adducts in mouse lines with gene-technically modified SULT1A1 status: comparison of various test compounds

Hansruedi Glatt [1,2,*], Sarah Yasmin Weißenberg [1], Anke Ehlers [1], Alfonso Lampen [1], Albrecht Seidel [3], Fabian Schumacher [2,4,†], Wolfram Engst [2,†] and Walter Meinl [2]

1 Department Food Safety, Federal Institute of Risk Assessment (BfR), Max-Dohrn-Strasse 8–10, 10589 Berlin, Germany; sarah.weissenberg@live.com (S.Y.W.); anke.ehlers@bfr.bund.de (A.E.); alfonso.lampen@bfr.bund.de (A.L.)
2 Department of Nutritional Toxicology, German Institute of Human Nutrition (DIfE), Potsdam-Rehbrücke, Arthur-Scheunert-Allee 114–116, 14558 Nuthetal, Germany; fabian.schumacher@fu-berlin.de (F.S.); wolfram.engst@web.de (W.E.); meinl@dife.de (W.M.)
3 Biochemical Institute for Environmental Carcinogens (BIU), Prof. Dr. Gernot Grimmer-Foundation, Lurup 4, 22927 Grosshansdorf, Germany; albrecht.seidel@biu-grimmer.de
4 Institute of Pharmacy, Freie Universität Berlin, Königin-Luise-Strasse 2–4, 14195 Berlin, Germany

* Correspondence: glatt@dife.de; Tel.: +49-30-691-6846

† During the time of the conduction of the study in DIfE.

1. Abbreviations: AAI, aristolochic acid I; AAII, aristolochic acids II; ABP, 4-aminobiphenyl; AhR, arylhydrocarbon receptor; BfR, Federal Institute of Risk Assessment; BIU, Biochemical Institute for Environmental Carcinogens; DIfE, German Institute of Human Nutrition; dN, 2-deoxynucleoside; FFA, furfuryl alcohol; γH2AX, phosphorylation of the histone protein H2AX; HMF, 5-hydroxymethylfurfural; 1-HMP, 1-hydroxymethylpyrene; i.p., intraperitoneal; LOD, limit of detection; ko, knockout of the *Sult1a1*; LC-MS/MS, liquid chromatography coupled with tandem mass spectrometry; ME, methyleugenol; 1-MIM-OH, 1-methoxy-3-indolylmethyl alcohol; 1-MP, 1-methylpyrene; MRM, multiple reaction monitoring; MS, mass spectrometry; *m*/*z*, mass-to-charge ratio; NAT, N-acetyltransferases; 3-NBA, 3-nitrobenzanthrone; OAT, organic anion transporter; PhIP, 2-amino-1-methyl-6-phenylimidazo[4,5-*b*]pyridine; PL, detection of DNA adducts using ^{32}P-postlabelling combined with multi-directional thin-layer chromatography: SCE, sister chromatid exchange; SMF, 5-sulfooxymethylfurfural; 1-SMP, 1-sulfooxymethylpyrene; SULT, sulfotransferase (human or generic; italics for genes romans for proteins and RNAs); Sult, sulfotransferase (mouse forms; italics for genes, romans for proteins and RNAs); SULT1A1/2, human SULT1A1 and/or SULT1A2 enzyme; tg, transgenic for the human *SULT1A1-SULT1A2* gene cluster; wt, wild-type.

2. Introduction: We have investigated a total of twelve compounds for DNA adduct formation and or other toxicological effects in ko, ko-tg, and/or tg compared to wt, mice – in some cases with additional knockout of Sult1d1 (which has no functional orthologue in humans). All these compounds had demonstrated enhanced mutagenicity in *Salmonella typhimurium* strains and or Chinese hamster V79 cells after expression of human SULT1A1 (Tables S1 & S2). Eleven compounds are known to have carcinogenic activity in animal models and/or are proximate genotoxicants of known carcinogens. 1-MIM-OH (the compound investigated in the present study) has not been studied for tumorigenesis in animals, either However, when tg mice were treated sub-chronically (for 90 d) with 1-MIM-OH, hepatic adduct levels accumulated over the time and 1-MIM-OH induced a gene expression profile similar to the expression signature caused by known genotoxic hepatocarcinogens [73].

The following compounds were studied:

- 1-Methoxy-3-indolylmethylalcohol (1-MIM-OH) – see present study
- 2-Amino-1-methyl-6-phenylimidazo[4,5-*b*]pyridine (PhIP): a heterocyclic aromatic amine, mainly formed in heated muscle meat
- 4-Aminobiphenyl (ABP): industrial chemical, also present in cigarette smoke, classical bladder carcinogen in humans
- 3-Nitrobenzanthrone (3-NBA): environmental pollutant
- Aristolochic acids I and II (AAI and AAII): secondary plant metabolites present in *Aristolochia* species; potent nephrocarcinogens in humans and animal models
- Methyleugenol (ME): secondary plant metabolite found in many foods and herbs, *e.g.,* at high levels in basil
- 1′-Hydroxymethyleugenol (1′-HME): major metabolite of ME
- 1-Methylpyrene (1-MP): common alkylated polycyclic aromatic hydrocarbon, *e.g.* present at high levels in cigarette smoke
- 1-Hydroxymethylpyrene (1-HMP): Major metabolite of 1-MP. The analysis of DNA adducts in animals treated with 1-methylpyrene demonstrated activation via 1-HMP and subsequent sulfation [77]
- Furfuryl alcohol (FFA): important intermediate in the heat-induced degradation of pentoses, glucose and fructose; pollutant in air resulting from the use of furan resins
- 5-Hydroxymethylfurfural (HMF), formed from hexoses treated with acid or heat, present at high levels in numerous foodstuffs, rather weak carcinogenic effects in several animal studies

We only present data for tissues studied with 1-MIM-OH in genetically modified mouse strains, *i.e.* liver, stomach, small intestine, caecum, colon, kidney and bone marrow (as comparison with the effects of 1-MIM-OH is the subject of this supplement). 1-MIM-OH is the only compound tested in bone marrow (and therefore bone marrow is ignored for the subsequent comparisons).

3. Effect of SULT1A1 and other SULT/NAT enzymes on genotoxic activities *in vitro*: Human SULT1A1 had enhanced the mutagenicity of all test compounds (or their phase I metabolites, *i.e.*, the proximate mutagens) in *S. typhimurium* (Ames test, using strains expressing SULTs) and or Chinese hamster V79 cells (gene mutations at *hprt* locus, SCE and/or DNA adducts). Here we primarily list compounds tested with additional human SULT forms, mouse Sults (in particular Sult1a1) or, human *N*-acetyltransferases (NATs, enzymes mediating the activation reactions of various aromatic hydroxylamines, analogously to the esterification mediated by SULTs).

Taken together the data (Tables S1 & S2) demonstrate that SULT1A1 is not the only human enzyme able to activate the compounds. However, the number of enzyme, the enzyme forms involved and their relative activities vary substantially among the set of compounds studied. In particular, many compounds that were activated by SULT1A1 were also toxified very efficiently by SULT1C2 (SULT1C4 in a newer nomenclature proposed). However, it appears that expression of this form is restricted to the foetal period [78].

Mouse Sult1a1 and human SULT1A1 showed similar activities in the bioactivation of various promutagens [(+)-1′-OH-ME, (–)-1′-OH-ME, 1-HMP, FFA and HMF]; however, the Sult1a1 showed negligible activity toward *N*-OH-PhIP (and 2-hydrox-3-methylcholanthrene [79]), two relatively large molecules, although the human orthologue was an excellent activator of these compounds (Table S1). Interestingly, Sult1d1 was rather active in the activation of many compounds of this list. Sult1d1 has no functional orthologue in humans. We have constructed and used a Sult1d1-knockout mouse line [80].

4. Synthesis and activities of sulfo conjugates: We have synthesised reactive sulfo conjugates of three compounds listed above, 1-HMP, FFA and HMF. Their half-life times in water at 37 °C were nearly 114 min, 20 s and 2.9 min respectively [81]. The corresponding sulfo conjugates of the other compounds have not been prepared in pure form in our or any other laboratories – possibly owing to very high reactivity.

1-Sulfooxymethylpyrene (1-SMP) and 5-sulfooxymethylfurfural (SMF) were detected in blood of animals treated with 1-HMP, and HMF, respectively [77, 80, 82]. They are substrates for the organic anion transporters (OAT) 1 and 3 [83, 84], mediating cellular uptake and are highly expressed at the basolateral (blood) site of proximal tubule cells. Indeed, direct intraperitoneal administration of these metabolites led to the formation of high levels of DNA adducts in kidney (1-SMP) [85] and massive damage to proximal tubule cells (SMF) [86].

Table S1: Effect of expression of SULTs and NATs in *Salmonella typhimurium* target cells on the mutagenicity of test compounds.

Compound	Starting strain	Revertants/nmol			
		Starting strain (no heterologous enzyme)	HumanSULT1A1 expressed	Mouse Sult1a1 expressed	Other SULT or NAT enzymes expressed
1-MIM-OH	*S. typhimurium* TA100 [51, 52]	3 [a]	600 [a]	–	1C2 (4,200)<br>inactive: 1A2, 1A3, 1E1, 2A1, 2B1b
(+)-1′-OH-ME	*S. typhimurium* TA100 [87]	< 0.5 [a]	12 [a] (35[b])	5	1A2 (0.7), 1C2 (12) 1E1 (1.5)<br>inactive: 1A3, 1C1, 1C3, 2A1, 2B1b, 1d1
(–)-1′-OH-ME	*S. typhimurium* TA100 [87]	< 0.5 [a]	8 [a] (25[b])	2	1A2 (0.4), 1C2 (5) 1E1 (1)<br>inactive: 1A3, 1C1, 1C3, 2A1, 2B1b, 1d1
N-OH-PhIP	*S. typhimurium* TA1538-DNP [c] [88]	10,400	102,100	–	inactive: NAT1, NAT2
N-OH-PhIP	*S. typhimurium* TA1538 [89]	18,000	480,000	≤ 18,000	1A2 (145,000)<br>1d1 (76,000)<br>inactive: 1A3, 1C1, 1C2, 1C3, 1E1, 2A1, 2B1a, 2B1b, 4A1, 1a1
AAI+AAII (commercial mixture) [d]	*S. typhimurium* TA1538 [90]	0.18	0.75 (4.8[b])	–	1B1 (1.1)<br>inactive: 1A2, 1A3, 1C1, 1C2, 1C3, 1E1, 2A1, 2B1a, 2B1b, 4A1
1-HMP	*S. typhimurium* TA1538 [79, 80]	< 0.3	7,000 (16,000[b])	8,300	1A2 (400), 1A3 (60), 1B1 (90), 1C2 (800), 1C3 (2), 1E1 (14,000), 2A1 (350) 1b1 (15), 1d1 (30), 1e1 (15,000); 2a1 (150), 2a2 (70)<br>inactive: 1C1[e], 1c2[e], 2a3 [e], 5a1[e]
FFA	*S. typhimurium* TA100, mutations [81]	< 10	1,800	2,500	1A2 (500), 1A3 (20), 1C2 (70) 1E1 (30), 1d1 (200)<br>inactive: 2A1
HMF	*S. typhimurium* TA100, mutations [81]	< 10	100	190	1A3 (10), 1C2 (700) 1d1 (30)<br>inactive: 1A1, 1E1, 2A1

[a] DNA adduct formation (detected by LC-MS/MS) was increased, in parallel to the mutagenic effects.
[b] Data in parentheses refer to strains expressing human SULT1A1 at increased levels (strains TA1538-SULT1A1*1Y or TA100-SULT1A1*1Y).
[c] Strain TA1538-DNP lacks an endogenous acetyltransferase (an enzyme able to activate various aromatic hydroxylamines).
[d] Bioactivation by human SULT1A1 was later confirmed for AAI as well AAII tested separately.
[e] Some activation (weak, but unambiguous) by these enzymes was detected under modified experimental conditions [80].
– Not tested

Table S2: Effect of expression of SULTs and NATs in Chinese hamster V79 target cells on genotoxic effects of the test compounds.

Compound	Endpoint studied	Cell lines used, strength of effect
MIM-OH [51]	SCE	V79-hSULT1A1 >> V79 (> 30-fold)
	Cytotoxicity	V79-hSULT1A1 > V79 (≥ 10-fold)
PhIP [89]	Gene mutations (hprt)	Positive (lowest effective concentration): V79-hCYP1A2-hSULT1A1 (0.3 μM) > V79-hCYP1A2-hSULT1A2 (1 μM) > V79-hCYP1A2-hNAT2 (10 μM)<br>Negative (highest concentration used: 30 μM): V79-hCYP1A2, V79-hCYP1A2-hNAT1
PhIP [91]	γH2AX	Positive (lowest effective concentration): V79-hCYP1A2-hSULT1A1 (0.1 μM) > V79-hCYP1A2-hNAT2 (1 μM) = V79-hCYP1A2 (1 μM)
AAI+AAII [90] (commercial mixture)	Gene mutations (hprt)	V79-hSULT1A1 > V79p [a] (4-fold)<br>V79-hCYP2E1-hSULT1A1: without SULT inhibitor > with SULT inhibitor pentachlorophenol (5-fold)
3-NBA [92]	DNA adducts (PL)	V79-hNAT2 >> V79-hSULT1A1 > V79-hNAT1 > V79 (several conditions)
3-NBA [93]	DNA adducts (PL)	V79-hCYP1A2-hNAT2 ≥ V79-hCYP1A2-hSULT1A1 ≥ V79-hCYP1A2-hSULT1A2 ≥ V79-hCYP1A2-hNAT1 >> V79-hCYP1A2 > V79 (several conditions)

[a] V79p: vector control (puromycin-resistant)

Table S3: DNA adducts and other toxicological findings in mouse lines with gene-technically modified SULT1A1 status.

Compound	Treatment, analytics, reference	Tissue	DNA adduct level (DNA breaks in the case of HMF)				Comment
			wt	ko	tg	ko-tg	
1-MIM-OH	600 µmol/kg (106 mg/kg), i.p., 8 h, N^2-(1-MIM)-dG (LC-MS/MS), this study	Liver	High 1/7	↓↓↓ by 98.2 %	↑ 2-fold	↑ 2.1-fold	
		Stomach	High 3/7	=	=	=	
		Small intestine	Low 5/7	=	↑↑↑ 19.2-fold	↑↑↑ 23.1-fold	
		Caecum	High 2/7	↓↓↓ by 98.9 %	=	=	
		Colon	Intermediate 4/7)	↓↓↓ by 95.4 %	=	=	
		Kidney	Low 6/7	↓↓ by 70 %	↑↑↑ 9.8-fold	↑↑↑ 16.2-fold	
PhIP	90 mg/kg, oral gavage, 8 h, PL), [59]	Liver	Low 8/8		↑↑↑ 13-fold		Note that in both studies liver was the tissue with the lowest adduct level in wt mice, whereas it was the tissue with highest adduct level in tg mice.
		Small intestine (jejunum)	Moderate 2/8		=		
		Small intestine (ileum)	Moderate 3/8		=		
		Caecum	High 1/8		↑ 1.5-fold		
		Colon	Moderate 5/8		↑ 2.0-fold		
		Kidney	Moderate 4/8		↑ 1.6-fold		
PhIP	75 mg/kg, oral gavage, 3 h, LC-MS/MS) [94]	Liver	Low 5/5		↑↑↑ 14-fold [a]		
		Proximal small intestine	Moderate 2/5		↑ 3.5-fold [a]		
		Distal small intestine	Moderate 1/5		↑ 2.5-fold [a]		
		Colon	Moderate 3/5		↑ 3.0-fold [a]		
		Kidney	Low 4/5		↑ 2.5-fold [a]		
PhIP	90 mg/kg, oral gavage, 8 h, LC-MS/MS), manuscript in preparation	Liver	Low 4/5	=	↑↑↑ 6.5-fold	↑↑↑ 11-fold [b]	Knockout of Sult1d1 reduced the adduct formation in all four tissues listed in this table (sites of Sult1d1 expression in wt mice), but not in lung (no expression detected in wt)
		Small intestine	Moderate 1/5	=	↑ 1.8-fold	↑ 1.6-fold [b]	
		Colon	Moderate 2/5	=	=	=	
		Kidney	Low 3/5	=	↑ 1.8-fold	↑ 2.0-fold [b]	
ABP	20 mg/kg, i.p., 24 dG-C8-ABP (LC-MS/MS) [95]	Liver	High 2/2	↓↓↓ by 98 %			Further decrease in adducts in Sult1a1-Sult1d1 double-knockout mice

AAI	50 mg/kg, oral gavage, 24 h, PL), [96]	Liver	Low 6/8	=	=		Transgenic human SULT1A1/2 had no effect in the remaining tissues studied, either
		Glandular stomach	Low 4/8		=		
		Small intestine	Moderate 3/8	=	=		
		Colon	Low 8/8		=		
		Kidney	High 1/8	=	=		
AAII	50 mg/kg, oral gavage, 24 h, PL), [96]	Liver	Low 6/8	=	=		Transgenic human SULT1A1/2 had no effect in the remaining tissues studied, either
		Glandular stomach	Low 5/8		=		
		Small intestine	Moderate 2/8	=	=		
		Colon	Low 8/8		=		
		Kidney	High 1/8	=	=		
3-NBA	2 mg/kg, i.p., 24 h, PL), [96]	Liver	High 1/9		=		.
		Glandular stomach	Moderate 5/9		↑ 2.5-fold		
		Small intestine	Low 7/9		↑↑ 4.0-fold		
		Colon	High 2/9		↑ 6.1-fold		
		Kidney	Low 6/9		↑↑ 1.4-fold		
ME	280 µmol/kg (50 mg/kg), oral gavage, 6 h, N^2-MIE-dG (LC-MS/MS) [68]	Liver	High 2/4	↓↓↓ by 96.1 %	↑↑ 6.1-fold	↑↑ 5.1-fold	
		Stomach	High 3/4	=	=	=	
		Caecum	High 1/4	↓↓↓ < LOD, by >97.5 %	↑ 2.3-fold	=	
		Kidney	< LOD	< LOD	+++ >32-fold	+++ >37-fold	
1′-OH-ME	280 µmol/kg (54 mg/kg), i.p., 6 h, N^2-MIE-dG (LC-MS/MS) [58],	Liver	High 1/1	↓↓↓ by 99.2 %	↑↑↑ 8.9-fold	↑↑↑ 8.3-fold	
1-MP	oral gavage, 500 µmol/kg (108 mg/kg), 2 h, N^2-(1-MP)-dG (LC-MS/MS) [77]	Liver	High 1/3		↑↑ 4.4-fold		The dramatic increase in the renal DNA levels detected in tg versus wt mice was accompanied by a similar increase the in serum levels of the reactive metabolite, 1-SMP
		Kidney	Moderate 2/3		↑↑↑ 21-fold		

1-HMP	83 μmol/kg 19.3 (mg/kg), oral gavage, 1 h, N^2-(1-MP)-dG (LC-MS/MS) [80]	Liver	High<br>1/5	↓↓↓<br>by 89 %	↑↑↑<br>13-fold		The dramatic increase in the renal DNA levels detected in tg versus wt mice was accompanied by a similar increase the in serum levels of the reactive metabolite, 1-SMP
		Colon	Low<br>4/5	=	=		
		Kidney	Moderate<br>2/5	↓↓<br>by 65 %	↑↑↑<br>83-fold		
FFA	400 mg/kg, i.p., 1 h, N^2-MF-dG (LC-MS/MS) [97]	Liver	Moderate<br>2/5	↓↓↓, < LOD<br>by >92 %	=	↑↑<br>3.4-fold [b]	Sult1d1-knockout led to significant decreases in adduct formation in kidney and small intestine
		Small intestine	Moderate<br>4/5	=		↑↑↑<br>6-fold [b]	
		Colon	Moderate<br>1/5	↓↓↓, < LOD<br>by >88 %		= [b]	
		Kidney	Moderate<br>3/5	↓↓<br>by 80 %		↑↑<br>4.5-fold [b]	
FFA	400 mg/kg, i.p., 1 h, N^2-MF-dG (LC-MS/MS) [98]	Liver	Moderate<br>2/4		=	↑↑<br>4.4-fold [b]	This study confirms the results of the preceding study. In addition, adduct formation in wt and double-ko-tg mice were enhanced when ethanol or an inhibitor of alcohol dehydrogenase was co- administered with FFA
		Colon	Moderate<br>1/4			= [b]	
		Kidney	Moderate<br>3/4			↑↑<br>4.7-fold [b]	
FFA	250 mg/kg, oral gavage, 1 h, LC-MS/MS), [99]	Liver	Low (↑ [c])<br>2/5		= [a, c]		
		Proximal small intestine	Low (= [c])<br>4/5		↑↑<br>7-fold [a, c]		
		Distal small intestine	Low (= [c])<br>5/5		= [a, c]		
		Colon	Low (↑↑[c])<br>1/5		= [a, c]		
		Kidney	Low (= [c])<br>3/5		= [a, c]		
HMF	900-1300 mg/kg, oral gavage, 1 h, DNA breaks (alkaline single cell gel electro-phoresis) [100]	Liver	0		0		
		Colon	0		0		
		Kidney	0		+		

All data presented were obtained with male mice (aged 8-10 weeks) and refer to the treatment scheme indicated in column 2. The list is incomplete in as much findings in females and with other treatment regimens are omitted; likewise findings in tissues other than liver, stomach, small intestine, caecum, colon and kidney are omitted.
Column 4: numbers in parentheses (m/n): n, number of tissues investigated in that study (including tissues omitted in the table); m, rank with regard to adduct level among these tissues (1 = highest).
Columns 5-7: comparison with effect in wt mice; =, no statistical difference; ↓, ↓↓, ↓↓↓: moderate, strong and very strong decrease in effect, respectively (% decrease is given in the next line for readers preferring numbers); ↑, ↑↑, ↑↑↑: moderate, strong and very strong increase in effect (fold increase in next line). If an effect was not observed either in the wt or the compared mouse line, LOD was used for calculating the minimal % decrease or fold increase. Comet assay: 0, no effect compared to vehicle control.

[a] Animals homozygous for human SULT1A1/2 were used (whereas mice hemizygous for this transgene) were used in the other studies presented unless specified otherwise.

[b] Human SULT1A1/2 was expressed Sult1a1/1d1-double knockout mice.

[c] Only study in which the adducts analysed were also detected in vehicle treated control animals, suggesting an unintended exposure to FFA (a wide-spread contaminant, present for example in building materials).

Empty fields: not tested.

5. Discussion

AAI and AAII: It is concluded that SULT1A1 enzymes are unimportant for the activation of AAs in the mouse models used, although human SULT1A1 had demonstrated (moderate) activation *in vitro* (Tables S1 & S2).

PhIP: Transgenic SULT1A1/2 drastically enhanced DNA adduct formation in liver, and to lower extents in many other tissues, whereas Sult1a1-knockout had no effect on the adduct formation in any tissues. However, Sult1d1-knockout reduced the adduct formation – selectively in tissues with Sult1d1 expression (kidney, small intestine and colon). The findings agree with results obtained in recombinant *S. typhimurium* strain: human SULT1A1 and SULT1A2 as well as mouse Sult1d1 activated *N*-OH-PhIP to a mutagen, whereas no activation was observed with mouse Sult1a1.

3-NBA: Experiments with recombinant V79 cells indicate that phase II enzymes (SULT or NAT) are important for efficient activation of 3-NBA. The highest adduct formation was observed in the liver – it was not affected by transgenic SULT1A1/2, suggesting that endogenous enzymes (probably Nat and Sult enzymes) were present at levels sufficient for effective activation. These enzymes have not been specified, as experiments with any knockout models are missing. However, transgenic SULT1A1/2 enhanced the adduct formation in various extrahepatic tissues of tg mice.

4-ABP, ME (and 1′-OH-ME), 1-HMP, FFA, 1-MIM-OH: Sult1a1-knockout led to drastic decreases in the formation of DNA adducts (by 89-99.2 %), implying that it is the principal activator in this tissue. Apart from the liver, Sult1a1 is highly expressed in colon and caecum. Adduct formation by some of these compounds was also studied in these tissues of Sult1a1-knockout mice. Like in the liver, adduct levels in ko mice were drastically decreased (88-98.9 %) after treatment with MIM-OH (studied in caecum and colon), ME (caecum) and FFA (colon). However, adduct formation in colon by 1-HMP was unaffected by this knockout, suggesting that other enzymes were important – indeed numerous different human and mouse SULT forms were able to activate 1-HMP in vitro (Table S1). With all test compounds, Sult1a1-knockout had no (or low) impact on the DNA adduct formation in small intestine and stomach, tissues with very low Sult1a1 expression. Kidney takes an intermediate position, complicated by the observation that some reactive sulfo-conjugates (1-SMP and SMF) are distributed in the organism via the circulation and actively taken up (by OAT transporters) into proximal tubule cells (section 4). Such a transfer of reactive sulfo conjugates does not appear to occur to an appreciable extent in the case of 1-MIM-OH and ME, as inferred from the very low renal adduct formation in wt mice.

Sult1a1-knockout either decreased or did not affect the adduct formation in a given tissue by any compound studied, but never increased it. Likewise, transgenic SULT1A1/2 enhanced or did not affect adduction, but did not decrease it. However the impacts of these genetic manipulations were not simply mirror-inverted. Transgenic SULT1A1/2 affected more tissues (*e.g.* small intestine and kidney) and compounds (*e.g.* PhIP) than Sult1a1-knockout. This is due to a wider tissue distribution and differing (usually broader) substrate tolerance for human SULT1A1 plus SULT1A2 as compared to Sult1a1. However the mouse contains another enzyme, Sult1d1 (without a functional orthologue in humans) able to activate pro-genotoxicants. Its substrate tolerance strongly overlaps with those of SULT1A enzymes, and it differs in its tissue distribution from Sult1a1.

6. References (as in the main part of this publication)

1. Blazevic, I.; Montaut, S.; Burcul, F.; Olsen, C.E.; Burow, M.; Rollin, P.; Agerbirk, N., Glucosinolate structural diversity, identification, chemical synthesis and metabolism in plants. *Phytochemistry* **2020,** *169*, 112100. doi: 10.1016/j.phytochem.2019.112100
2. Fahey, J.W.; Zhang, Y.S.; Talalay, P., Broccoli sprouts: An exceptionally rich source of inducers of enzymes that protect against chemical carcinogens. *Proc. Natl. Acad. Sci. USA* **1997,** *94*, 10367-10372. doi: 10.1073/pnas.94.19.10367
3. Agerbirk, N.; Olsen, C.E., Glucosinolate structures in evolution. *Phytochemistry* **2012,** *77*, 16-45. doi: 10.1016/j.phytochem.2012.02.005
4. Clarke, D.B., Glucosinolates, structures and analysis in food. *Anal. Meth.* **2010,** *2*, 310-325. doi: 10.1039/B9AY00280D
5. Widemann, E.; Bruinsma, K.; Walshe-Roussel, B.; Rioja, C.; Arbona, V.; Saha, R.K.; Letwin, D.; Zhurov, V.; Gomez-Cadenas, A.; Bernards, M.A.; Grbic, M.; Grbic, V., Multiple indole glucosinolates and myrosinases defend Arabidopsis against *Tetranychus urticae* herbivory. *Plant Physiol.* **2021,** *187*, 116-132. doi: 10.1093/plphys/kiab247
6. Aires, A.; Mota, V.R.; Saavedra, M.J.; Monteiro, A.A.; Simoes, M.; Rosa, E.A.; Bennett, R.N., Initial *in vitro* evaluations of the antibacterial activities of glucosinolate enzymatic hydrolysis products against plant pathogenic bacteria. *J. Appl. Microbiol.* **2009,** *106*, 2096-2105. doi: 10.1111/j.1365-2672.2009.04181.x
7. Lambrix, V.; Reichelt, M.; Mitchell-Olds, T.; Kliebenstein, D.J.; Gershenzon, J., The Arabidopsis epithiospecifier protein promotes the hydrolysis of glucosinolates to nitriles and influences Trichoplusia ni herbivory. *Plant Cell* **2001,** *13*, 2793-807. doi: 10.1105/tpc.010261

8. Opitz, S.E.; Mix, A.; Winde, I.B.; Muller, C., Desulfation followed by sulfation: metabolism of benzylglucosinolate in Athalia rosae (Hymenoptera: Tenthredinidae). *Chembiochem* **2011,** *12*, 1252-7. doi: 10.1002/cbic.201100053
9. Sontowski, R.; Guyomar, C.; Poeschl, Y.; Weinhold, A.; van Dam, N.M.; Vassao, D.G., Mechanisms of isothiocyanate detoxification in larvae of two Bbelowground herbivores, *Delia radicum* and *D. floralis* (Diptera: Anthomyiidae). *Front. Physiol.* **2022,** *13*, 874527. doi: 10.3389/fphys.2022.874527
10. Agerbirk, N.; Olsen, C.E.; Sørensen, H., Initial and final products, nitriles, and ascorbigens produced in myrosinase-catalyzed hydrolysis of indole glucosinolates. *J. Agr. Food Chem.* **1998,** *46*, 1563-1571. doi: org/10.1021/jf9708498
11. Hanschen, F.S.; Platz, S.; Mewis, I.; Schreiner, M.; Rohn, S.; Kroh, L.W., Thermally induced degradation of sulfur-containing aliphatic glucosinolates in broccoli sprouts (*Brassica oleracea* var. *italica*) and model systems. *J. Agric. Food Chem.* **2012,** *60*, 2231-2241. doi: 10.1021/jf204830p
12. Verkerk, R.; Schreiner, M.; Krumbein, A.; Ciska, E.; Holst, B.; Rowland, I.; De Schrijver, R.; Hansen, M.; Gerhäuser, C.; Mithen, R.; Dekker, M., Glucosinolates in *Brassica* vegetables: the influence of the food supply chain on intake, bioavailability and human health. *Mol. Nutr. Food Res.* **2009,** *53 Suppl 2*, S219-S265. doi: 10.1002/mnfr.200800065
13. Kumar, A.; Sabbioni, G., New biomarkers for monitoring the levels of isothiocyanates in humans. *Chem. Res. Toxicol.* **2009,** *23*, 756-765. doi: 10.1021/tx900393t
14. Kumar, A.; Vineis, P.; Sacerdote, C.; Fiorini, L.; Sabbioni, G., Determination of new biomarkers to monitor the dietary consumption of isothiocyanates. *Biomarkers* **2010,** *15*, 739-745. doi: 10.3109/1354750X.2010.517567
15. Barknowitz, G.; Engst, W.; Schmidt, S.; Bernau, M.; Monien, B.H.; Kramer, M.; Florian, S.; Glatt, H.R., Identification and quantification of protein adducts formed by metabolites of 1-methoxy-3-indolylmethyl glucosinolate *in vitro* and in mouse models. *Chem. Res. Toxicol.* **2014,** *27*, 188-199. doi: 10.1021/tx400277w
16. Wiesner-Reinhold, M.; Barknowitz, G.; Florian, S.; Mewis, I.; Schumacher, F.; Schreiner, M.; Glatt, H.R., 1-Methoxy-3-indolylmethyl DNA adducts in six tissues, and blood protein adducts, in mice under pak choi diet: time course and persistence. *Arch. Toxicol.* **2019,** *93*, 1515-1527. doi: 10.1007/s00204-019-02452-3
17. Barknowitz, G. Serumalbumin- und Hämoglobin-Addukte als Biomarker der Exposition gegenüber mutagenen Metaboliten von 1-Methoxy-3-indolylmethyl-glucosinolat – Untersuchungen in Maus und Mensch. Ph. D. thesis, University of Potsdam, Germany, Potsdam, 2013.
18. Kassie, F.; Parzefall, W.; Musk, S.; Johnson, I.; Lamprecht, G.; Sontag, G.; Knasmüller, S., Genotoxic effects of crude juices from *Brassica* vegetables and juices and extracts from phytopharmaceutical preparations and spices of cruciferous plants origin in bacterial and mammalian cells. *Chem.-Biol. Interact.* **1996,** *102*, 1-16. doi: 10.1016/0009-2797(96)03728-3
19. Wiesner, M.; Schreiner, M.; Glatt, H.R., High mutagenic activity of juice from pak choi (*Brassica rapa* ssp. *chinensis*) sprouts due to its content of 1-methoxy-3-indolylmethyl glucosinolate, and its enhancement by elicitation with methyl jasmonate. *Food Chem. Toxicol.* **2014,** *67*, 10-16. doi: 10.1016/j.fct.2014.02.008
20. Musk, S.R.; Johnson, I.T., The clastogenic effects of isothiocyanates. *Mutat. Res.* **1993,** *300*, 111-7. doi: 10.1016/0165-1218(93)90128-z
21. Musk, S.R.; Smith, T.K.; Johnson, I.T., On the cytotoxicity and genotoxicity of allyl and phenethyl isothiocyanates and their parent glucosinolates sinigrin and gluconasturtiin. *Mutat. Res.* **1995,** *348*, 19-23. doi: 10.1016/0165-7992(95)90016-0
22. Musk, S.R.R.; Astley, S.B.; Edwards, S.M.; Stephenson, P.; Hubert, R.B.; Johnson, I.T., Cytotoxic and clastogenic effects of benzyl isothiocyanate towards cultured mammalian cells. *Food Chem. Toxicol.* **1995,** *33*, 31-37. doi: 10.1016/0278-6915(95)80245-2
23. Yamaguchi, T., Mutagenicity of isothiocyanates, isocyanates and thioureas on *Salmonella typhimurium*. *Agric. Biol. Chem.* **1980,** *44*, 3017-3018. doi: org/10.1080/00021369.1980.10864451
24. Neudecker, T.; Henschler, D., Allyl isothiocyanate is mutagenic in *Salmonella typhimurium*. *Mutat. Res.* **1985,** *156*, 33-37. doi: 10.1016/0165-1218(85)90004-7
25. Kassie, F.; Knasmüller, S., Genotoxic effects of allyl isothiocyanate (AITC) and phenethyl isothiocyanate (PEITC). *Chem.-Biol. Interact.* **2000,** *127*, 163-180. doi: 10.1016/s0009-2797(00)00178-2
26. Kassie, F.; Pool-Zobel, B.; Parzefall, W.; Knasmüller, S., Genotoxic effects of benzyl isothiocyanate, a natural chemopreventive agent. *Mutagenesis* **1999,** *14*, 595-603. doi: 10.1093/mutage/14.6.595
27. Baasanjav-Gerber, C.; Monien, B.H.; Mewis, I.; Schreiner, M.; Barillari, J.; Iori, R.; Glatt, H.R., Identification of glucosinolate congeners able to form DNA adducts and to induce mutations upon activation by myrosinase. *Mol. Nutr. Food Res.* **2011,** *55*, 783-792. doi: 10.1002/mnfr.201000352
28. Latté, K.P.; Appel, K.E.; Lampen, A., Health benefits and possible risks of broccoli: an overview. *Food Chem. Toxicol.* **2011,** *49*, 3287-3309. doi: 10.1016/j.fct.2011.08.019
29. van Poppel, G.; Verhoeven, D.T.; Verhagen, H.; Goldbohm, R.A., *Brassica* vegetables and cancer prevention: epidemiology and mechanisms. *Adv. Exp. Med. Biol.* **1999,** *472*, 159-168. doi: 10.1007/978-1-4757-3230-6_14
30. Verhoeven, D.T.; Verhagen, H.; Goldbohm, R.A.; van den Brandt, P.A.; van Poppel, G., A review of mechanisms underlying anticarcinogenicity by *Brassica* vegetables. *Chem.-Biol. Interact.* **1997,** *103*, 79-129. doi: 10.1016/s0009-2797(96)03745-3.
31. Dinkova-Kostova, A.T.; Kostov, R.V., Glucosinolates and isothiocyanates in health and disease. *Trends Mol. Med.* **2012,** *18*, 337-347. doi: 10.1016/j.molmed.2012.04.003

32. Wu, Q.J.; Xie, L.; Zheng, W.; Vogtmann, E.; Li, H.L.; Yang, G.; Ji, B.T.; Gao, Y.T.; Shu, X.O.; Xiang, Y.B., Cruciferous vegetables consumption and the risk of female lung cancer: a prospective study and a meta-analysis. *Ann. Oncol.* **2013,** *24*, 1918-1924. doi: 10.1093/annonc/mdt119
33. Wu, Q.J.; Yang, Y.; Vogtmann, E.; Wang, J.; Han, L.H.; Li, H.L.; Xiang, Y.B., Cruciferous vegetables intake and the risk of colorectal cancer: a meta-analysis of observational studies. *Ann. Oncol.* **2013,** *24*, 1079-1087. doi: 10.1093/annonc/mds601
34. Wu, Q.J.; Yang, Y.; Wang, J.; Han, L.H.; Xiang, Y.B., Cruciferous vegetable consumption and gastric cancer risk: a meta-analysis of epidemiological studies. *Cancer Sci.* **2013,** *104*, 1067-1073. doi: 10.1111/cas.12195
35. Bonnesen, C.; Eggleston, I.M.; Hayes, J.D., Dietary indoles and isothiocyanates that are generated from cruciferous vegetables can both stimulate apoptosis and confer protection against DNA damage in human colon cell lines. *Cancer Res.* **2001,** *61*, 6120-6130. doi: PMID: 11507062
36. Kensler, T.W.; Egner, P.A.; Agyeman, A.S.; Visvanathan, K.; Groopman, J.D.; Chen, J.-G.; Chen, T.-Y.; Fahey, J.W.; Talalay, P., Keap1-Nrf2 signaling: a target for cancer prevention by sulforaphane. *Top. Curr. Chem.* **2013,** *329*, 163-177. doi: 10.1007/128_2012_339
37. Bonnesen, C.; Stephensen, P.U.; Andersen, O.; Sorensen, H.; Vang, O., Modulation of cytochrome P-450 and glutathione S-transferase isoform expression in vivo by intact and degraded indolyl glucosinolates. *Nutr. Cancer* **1999,** *33*, 178-87. doi: 10.1207/S15327914NC330210
38. Zhang, Y.; Talalay, P.; Cho, C.G.; Posner, G.H., A major inducer of anticarcinogenic protective enzymes from broccoli: isolation and elucidation of structure. *Proc. Natl. Acad. Sci. USA* **1992,** *89*, 2399-2403. doi: 10.1073/pnas.89.6.2399
39. Higgins, L.G.; Kelleher, M.O.; Eggleston, I.M.; Itoh, K.; Yamamoto, M.; Hayes, J.D., Transcription factor Nrf2 mediates an adaptive response to sulforaphane that protects fibroblasts *in vitro* against the cytotoxic effects of electrophiles, peroxides and redox-cycling agents. *Toxicol. Appl. Pharmacol.* **2009,** *237*, 267-280. doi: 10.1016/j.taap.2009.03.005
40. McWalter, G.K.; Higgins, L.G.; McLellan, L.I.; Henderson, C.J.; Song, L.; Thornalley, P.J.; Itoh, K.; Yamamoto, M.; Hayes, J.D., Transcription factor Nrf2 is essential for induction of NAD(P)H:quinone oxidoreductase 1, glutathione S-transferases, and glutamate cysteine ligase by broccoli seeds and isothiocyanates. *J. Nutr.* **2004,** *134*, 3499S-3506S. doi: 10.1093/jn/134.12.3499S
41. Bjeldanes, L.F.; Kim, J.Y.; Grose, K.R.; Bartholomew, J.C.; Bradfield, C.A., Aromatic hydrocarbon responsiveness-receptor agonists generated from indole-3-carbinol *in vitro* and *in vivo*: comparisons with 2,3,7,8-tetrachlorodibenzo-*p*-dioxin. *Proc. Natl. Acad. Sci. USA* **1991,** *88*, 9543-9547. doi: 10.1073/pnas.88.21.9543
42. Williams, D.E., Indoles derived from glucobrassicin: Cancer chemoprevention by indole-3-carbinol and 3,3′-diindolylmethane. *Front. Nutr.* **2021,** *8*, 734334. doi: 10.3389/fnut.2021.734334
43. Bock, K.W.; Köhle, C., Ah receptor: Dioxin-mediated toxic responses as hints to deregulated physiologic functions. *Biochem. Pharmacol.* **2006,** *72*, 393-404. doi: org/10.1016/j.bcp.2006.01.017
44. Diaz-Diaz, C.J.; Ronnekleiv-Kelly, S.M.; Nukaya, M.; Geiger, P.G.; Balbo, S.; Dator, R.; Megna, B.W.; Carney, P.R.; Bradfield, C.A.; Kennedy, G.D., The aryl hydrocarbon receptor is a repressor of inflammation-associated colorectal tumorigenesis in mouse. *Ann. Surg.* **2016,** *264*, 429-436. doi: 10.1097/SLA.0000000000001874
45. Peng, C.; Wu, C.; Xu, X.; Pan, L.; Lou, Z.; Zhao, Y.; Jiang, H.; He, Z.; Ruan, B., Indole-3-carbinol ameliorates necroptosis and inflammation of intestinal epithelial cells in mice with ulcerative colitis by activating aryl hydrocarbon receptor. *Exp. Cell Res.* **2021,** *404*, 112638. doi: 10.1016/j.yexcr.2021.112638
46. Gronke, K.; Hernandez, P.P.; Zimmermann, J.; Klose, C.S.N.; Kofoed-Branzk, M.; Guendel, F.; Witkowski, M.; Tizian, C.; Amann, L.; Schumacher, F.; Glatt, H.; Triantafyllopoulou, A.; Diefenbach, A., Interleukin-22 protects intestinal stem cells against genotoxic stress. *Nature* **2019,** *566*, 249-253. doi: 10.1038/s41586-019-0899-7
47. Fiala, J.L.; Egner, P.A.; Wiriyachan, N.; Ruchirawat, M.; Kensler, K.H.; Wogan, G.N.; Groopman, J.D.; Croy, R.G.; Essigmann, J.M., Sulforaphane-mediated reduction of aflatoxin B(1)-N(7)-guanine in rat liver DNA: impacts of strain and sex. *Toxicol. Sci.* **2011,** *121*, 57-62. doi: 10.1093/toxsci/kfr026
48. Dashwood, R.H.; Arbogast, D.N.; Fong, A.T.; Pereira, C.; Hendricks, J.D.; Bailey, G.S., Quantitative inter-relationships between aflatoxin B1 carcinogen dose, indole-3-carbinol anti-carcinogen dose, target organ DNA adduction and final tumor response. *Carcinogenesis* **1989,** *10*, 175-81. doi: 10.1093/carcin/10.1.175
49. Hecht, S.S.; Kenney, P.M.; Wang, M.; Upadhyaya, P., Benzyl isothiocyanate: an effective inhibitor of polycyclic aromatic hydrocarbon tumorigenesis in A/J mouse lung. *Cancer Lett.* **2002,** *187*, 87-94. doi: 10.1016/s0304-3835(02)00410-x
50. Xu, C.J.; Huang, M.T.; Shen, G.X.; Yuan, X.L.; Lin, W.; Khor, T.O.; Conney, A.H.; Kong, A.N.T., Inhibition of 7,12-dimethylbenz[*a*]anthracene-induced skin tumorigenesis in C57BL/6 mice by sulforaphane is mediated by nuclear factor E2-related factor 2. *Cancer Res.* **2006,** *66*, 8293-8296. doi: 10.1158/0008-5472.can-06-0300
51. Glatt, H.R.; Baasanjav-Gerber, C.; Schumacher, F.; Monien, B.H.; Schreiner, M.; Frank, H.; Seidel, A.; Engst, W., 1-Methoxy-3-indolylmethyl glucosinolate: a potent genotoxicant in bacterial and mammalian cells: Mechanisms of bioactivation. *Chem.-Biol. Interact.* **2011,** *192*, 81-6. doi: 10.1016/j.cbi.2010.09.009
52. Baasanjav-Gerber, C. Detection and identification of genotoxicants from *Brassica* plants. Ph.D. thesis, University of Potsdam, Germany, Potsdam, 2011.
53. Glatt, H.R.; Engst, W.; Florian, S.; Schreiner, M.; Baasanjav-Gerber, C., Feeding *Brassica* vegetables to rats leads to the formation of characteristic DNA adducts (from 1-methoxy-3-indolylmethyl glucosinolate) in many tissues. *Arch. Toxicol.* **2022,** *96*, 933-944. doi: 10.1007/s00204-021-03216-8

54. Schumacher, F.; Florian, S.; Schnapper, A.; Monien, B.H.; Mewis, I.; Schreiner, M.; Seidel, A.; Engst, W.; Glatt, H.R., A secondary metabolite of Brassicales, 1-methoxy-3-indolylmethyl glucosinolate, as well as its degradation product, 1-methoxy-3-indolylmethyl alcohol, forms DNA adducts in the mouse, but in varying tissues and cells. *Arch. Toxicol.* **2014,** *88*, 823-836. doi: 10.1007/s00204-013-1149-7
55. Hanley, A.B.; Parsley, K.R., Identification of 1-methoxyindolyl-3-methyl isothiocyanate as an indole glucosinolate breakdown product. *Phytochemistry* **1990,** *29*, 769-771. doi: org/10.1016/0031-9422(90)80015-9
56. Hanley, A.B.; Parsley, K.R.; Lewis, J.A.; Fenwick, R.G., Chemistry of indole glucosinolates: intermediacy of indol-3-ylmethyl isothiocyanates in the enzymic hydrolysis of indole glucosinoates. *J. Chem. Soc. Perkin Trans.* **1990**, 2273-2276. doi: 10.1039/P19900002273
57. Blanchard, R.L.; Freimuth, R.R.; Buck, J.; Weinshilboum, R.M.; Coughtrie, M.H., A proposed nomenclature system for the cytosolic sulfotransferase (SULT) superfamily. *Pharmacogenetics* **2004,** *14*, 199-211. doi: 10.1097/00008571-200403000-00009
58. Herrmann, K.; Engst, W.; Meinl, W.; Florian, S.; Cartus, A.T.; Schrenk, D.; Appel, K.E.; Nolden, T.; Himmelbauer, H.; Glatt, H.R., Formation of hepatic DNA adducts by methyleugenol in mouse models: drastic decrease by Sult1a1 knockout and strong increase by transgenic human SULT1A1/2. *Carcinogenesis* **2014,** *35*, 935-941. doi: 10.1093/carcin/bgt408
59. Dobbernack, G.; Meinl, W.; Schade, N.; Florian, S.; Wend, K.; Voigt, I.; Himmelbauer, H.; Gross, M.; Liehr, T.; Glatt, H.R., Altered tissue distribution of 2-amino-1-methyl-6-phenylimidazo[4,5-*b*]pyridine-DNA adducts in mice transgenic for human sulfotransferases 1A1 and 1A2. *Carcinogenesis* **2011,** *32*, 1734-1740. doi: 10.1093/carcin/bgr204
60. Schumacher, F.; Engst, W.; Monien, B.H.; Florian, S.; Schnapper, A.; Steinhäuser, L.; Albert, K.; Frank, H.; Seidel, A.; Glatt, H.R., Detection of DNA adducts originating from 1-methoxy-3-indolylmethyl glucosinolate using isotope-dilution UPLC-ESI-MS/MS. *Anal. Chem.* **2012,** *84*, 6256-6062. doi: 10.1021/ac301436q
61. Alnouti, Y.; Klaassen, C.D., Tissue distribution and ontogeny of sulfotransferase enzymes in mice. *Toxicol. Sci.* **2006,** *93*, 242-255. doi: 10.1093/toxsci/kfl050
62. Landsiedel, R.; Engst, W.; Scholtyssek, M.; Seidel, A.; Glatt, H.R., Benzylic sulphuric acid esters react with diverse functional groups and often form secondary reactive species. *Polycycl. Aromat. Compd.* **1996,** *11*, 341-348. doi: org/10.1080/10406639608544685
63. Hayashi, M., The micronucleus test-most widely used i*n vivo* genotoxicity test. *Genes Environ.* **2016,** *38*, 18. doi: 10.1186/s41021-016-0044-x
64. Sommer, S.; Buraczewska, I.; Kruszewski, M., Micronucleus assay: The state of art, and future directions. *Int. J. Mol. Sci.* **2020,** *21*. doi: 10.3390/ijms21041534
65. Glatt, H.R., Sulfotransferases in the bioactivation of xenobiotics. *Chem.-Biol. Interact.* **2000,** *129*, 141-70. doi: 10.1016/s0009-2797(00)00202-7
66. Riches, Z.; Stanley, E.L.; Bloomer, J.C.; Coughtrie, M.W., Quantitative evaluation of the expression and activity of five major sulfotransferases (SULTs) in human tissues: the SULT "pie". *Drug Metab. Dispos.* **2009,** *37*, 2255-2261. doi: 10.1124/dmd.109.028399
67. Teubner, W.; Meinl, W.; Florian, S.; Kretzschmar, M.; Glatt, H.R., Identification and localization of soluble sulfotransferases in the human gastrointestinal tract. *Biochem. J.* **2007,** *404*, 207-215. doi: 10.1042/BJ20061431
68. Herrmann, K.; Engst, W.; Florian, S.; Lampen, A.; Meinl, W.; Glatt, H.R., The influence of the SULT1A status – wild-type, knockout or humanized – on the DNA adduct formation by methyleugenol in extrahepatic tissues of mice. *Toxicol. Res.* **2016,** *5*, 808-815. doi: 10.1039/c5tx00358j
69. Riboli, E.; Beland, F.A.; Lachenmeier, D.W.; Marques, M.M.; Phillips, D.H.; Schernhammer, E.; Afghan, A.; Assuncao, R.; Caderni, G.; Corton, J.C.; de Aragao Umbuzeiro, G.; de Jong, D.; Deschasaux-Tanguy, M.; Hodge, A.; Ishihara, J.; Levy, D.D.; Mandrioli, D.; McCullough, M.L.; McNaughton, S.A.; Morita, T.; Nugent, A.P.; Ogawa, K.; Pandiri, A.R.; Sergi, C.M.; Touvier, M.; Zhang, L.; Benbrahim-Tallaa, L.; Chittiboyina, S.; Cuomo, D.; DeBono, N.L.; Debras, C.; de Conti, A.; El Ghissassi, F.; Fontvieille, E.; Harewood, R.; Kaldor, J.; Mattock, H.; Pasqual, E.; Rigutto, G.; Simba, H.; Suonio, E.; Viegas, S.; Wedekind, R.; Schubauer-Berigan, M.K.; Madia, F., Carcinogenicity of aspartame, methyleugenol, and isoeugenol. *Lancet Oncol.* **2023,** *24*, 848-850. doi: 10.1016/S1470-2045(23)00341-8
70. Miller, E.C.; Swanson, A.B.; Phillips, D.H.; Fletcher, T.L.; Liem, A.; Miller, J.A., Structure-activity studies of the carcinogenicities in the mouse and rat of some naturally occurring and synthetic alkenylbenzene derivatives related to safrole and estragole. *Cancer Res.* **1983,** *43*, 1124-1134. doi: PMID: 6825084
71. NTP, Toxicology and carcinogenesis studies of methyleugenol. *Natl. Toxicol. Program Tech. Rep. Ser.* **2000,** *491*, 1-420. doi: PMID: 12563349
72. Otteneder, M.; Lutz, W.K., Correlation of DNA adduct levels with tumor incidence: carcinogenic potency of DNA adducts. *Mutat. Res.* **1999,** *424*, 237-247. doi: 10.1016/s0027-5107(99)00022-6
73. Ehlers, A.; Florian, S.; Schumacher, F.; Meinl, W.; Lenze, D.; Hummel, M.; Heise, T.; Seidel, A.; Glatt, H.R.; Lampen, A., The glucosinolate metabolite 1-methoxy-3-indolylmethyl alcohol induces a gene expression profile in mouse liver similar to the expression signature caused by known genotoxic hepatocarcinogens. *Mol. Nutr. Food Res.* **2015,** *59*, 685-697. doi: 10.1002/mnfr.201400707
74. Tremmel, R.; Herrmann, K.; Engst, W.; Meinl, W.; Klein, K.; Glatt, H.R.; Zanger, U.M., Methyleugenol DNA adducts in human liver are associated with SULT1A1 copy number variations and expression levels. *Arch. Toxicol.* **2017,** *91*, 3329-3339. doi: 10.1007/s00204-017-1955-4

75. Monien, B.H.; Schumacher, F.; Herrmann, K.; Glatt, H.R.; Turesky, R.J.; Chesne, C., Simultaneous detection of multiple DNA adducts in human lung samples by isotope-dilution UPLC-MS/MS. *Anal. Chem.* **2015,** *87*, 641-648. doi: 10.1021/ac503803m
76. Vo, Q.V.; Trenerry, C.; Rochfort, S.; Wadeson, J.; Leyton, C.; Hughes, A.B., Synthesis and anti-inflammatory activity of indole glucosinolates. *Bioorg. Med. Chem.* **2014,** *22*, 856-864. doi: 10.1016/j.bmc.2013.12.003
77. Bendadani, C.; Meinl, W.; Monien, B.H.; Dobbernack, G.; Glatt, H.R., The carcinogen 1-methylpyrene forms benzylic DNA adducts in mouse and rat tissues *in vivo* via a reactive sulphuric acid ester. *Arch. Toxicol.* **2014,** *88*, 815-821. doi: 10.1007/s00204-013-1182-6
78. Sakakibara, Y.; Yanagisawa, K.; Katafuchi, J.; Ringer, D.P.; Takami, Y.; Nakayama, T.; Suiko, M.; Liu, M.C., Molecular cloning, expression, and characterization of novel human SULT1C sulfotransferases that catalyze the sulfonation of *N*-hydroxy-2-acetylaminofluorene. *J. Biol. Chem.* **1998,** *273*, 33929-33935. doi: 10.1074/jbc.273.51.33929
79. Meinl, W.; Tsoi, C.; Swedmark, S.; Tibbs, Z.E.; Falany, C.N.; Glatt, H.R., Highly selective bioactivation of 1- and 2-hydroxy-3-methylcholanthrene to mutagens by individual human and other mammalian sulphotransferases expressed in *Salmonella typhimurium*. *Mutagenesis* **2013,** *28*, 609-619. doi: 10.1093/mutage/get039
80. Bendadani, C.; Meinl, W.; Monien, B.H.; Dobbernack, G.; Florian, S.; Engst, W.; Nolden, T.; Himmelbauer, H.; Glatt, H.R., Determination of sulfotransferase forms involved in the metabolic activation of the genotoxicant 1-hydroxymethylpyrene using bacterially expressed enzymes and genetically modified mouse models. *Chem. Res. Toxicol.* **2014,** *27*, 1060-9. doi: 10.1021/tx500129g
81. Glatt, H.; Schneider, H.; Murkovic, M.; Monien, B.H.; Meinl, W., Hydroxymethyl-substituted furans: mutagenicity in Salmonella typhimurium strains engineered for expression of various human and rodent sulphotransferases. *Mutagenesis* **2012,** *27*, 41-8. doi: 10.1093/mutage/ger054
82. Monien, B.H.; Frank, H.; Seidel, A.; Glatt, H.R., Conversion of the common food constituent 5-hydroxymethylfurfural into a mutagenic and carcinogenic sulfuric acid ester in the mouse in vivo. *Chem. Res. Toxicol.* **2009,** *22*, 1123-1128. doi: 10.1021/tx9000623
83. Bakhiya, N.; Monien, B.H.; Frank, H.; Seidel, A.; Glatt, H.R., Renal organic anion transporters OAT1 and OAT3 mediate the cellular accumulation of 5-sulfooxymethylfurfural, a reactive, nephrotoxic metabolite of the Maillard product 5-hydroxymethylfurfural. *Biochem. Pharmacol.* **2009,** *78*, 414-419. doi: 10.1016/j.bcp.2009.04.017
84. Bakhiya, N.; Stephani, M.; Bahn, A.; Ugele, B.; Seidel, A.; Burckhardt, G.; Glatt, H.R., Uptake of chemically reactive, DNA-damaging sulfuric acid esters into renal cells by human organic anion transporters. *J. Amer. Soc. Nephrol.* **2006,** *17*, 1414-1421. doi: 10.1681/ASN.2005080801
85. Glatt, H.R.; Meinl, W.; Kuhlow, A.; Ma, L., Metabolic formation, distribution and toxicological effects of reactive sulphuric acid esters. *Nova Acta Leopoldina NF87* **2003,** *329*, 151-161.
86. Bauer-Marinovic, M.; Taugner, F.; Florian, S.; Glatt, H.R., Toxicity studies with 5-hydroxymethylfurfural and its metabolite 5-sulphooxymethylfurfural in wild-type mice and transgenic mice expressing human sulphotransferases 1A1 and 1A2. *Arch. Toxicol.* **2012,** *86*, 701-711. doi: 10.1007/s00204-012-0807-5
87. Herrmann, K.; Engst, W.; Appel, K.E.; Monien, B.H.; Glatt, H.R., Identification of human and murine sulfotransferases able to activate hydroxylated metabolites of methyleugenol to mutagens in *Salmonella typhimurium* and detection of associated DNA adducts using UPLC-MS/MS methods. *Mutagenesis* **2012,** *27*, 453-462. doi: 10.1093/mutage/ges004
88. Muckel, E.; Frandsen, H.; Glatt, H.R., Heterologous expression of human N-acetyltransferases 1 and 2 and sulfotransferase 1A1 in *Salmonella typhimurium* for mutagenicity testing of heterocyclic amines. *Food Chem. Toxicol.* **2002,** *40*, 1063-1068. doi: 10.1016/S0278-6915(02)00032-7
89. Glatt, H.R., Metabolic factors affecting the mutagenicity of heterocyclic amines. In *Acrylamide and Other Health Hazardous Compounds in Heat-treated Foods*, Skog, K.; Alexander, J., Eds. Woodhead Publishing: Cambridge, 2006; pp 358-404. doi: 10.1533/9781845692018.2.358
90. Meinl, W.; Pabel, U.; Osterloh-Quiroz, M.; Hengstler, J.G.; Glatt, H.R., Human sulfotransferases are involved in the activation of aristolochic acids and are expressed in renal target tissue. *Int. J. Cancer* **2006,** *118*, 1090-1097. doi: 10.1002/ijc.21480
91. Chevereau, M.; Glatt, H.; Zalko, D.; Cravedi, J.P.; Audebert, M., Role of human sulfotransferase 1A1 and N-acetyltransferase 2 in the metabolic activation of 16 heterocyclic amines and related heterocyclics to genotoxicants in recombinant V79 cells. *Arch Toxicol* **2017,** *91*, 3175-3184. doi: 10.1007/s00204-017-1935-8
92. Arlt, V.M.; Glatt, H.R.; Muckel, E.; Pabel, U.; Sorg, B.L.; Schmeiser, H.H.; Phillips, D.H., Metabolic activation of the environmental contaminant 3-nitrobenzanthrone by human acetyltransferases and sulfotransferase. *Carcinogenesis* **2002,** *23*, 1937-45. doi: 10.1093/carcin/23.11.1937
93. Arlt, V.M.; Glatt, H.R.; Muckel, E.; Pabel, U.; Sorg, B.L.; Seidel, A.; Frank, H.; Schmeiser, H.H.; Phillips, D.H., Activation of 3-nitrobenzanthrone and its metabolites by human acetyltransferases, sulfotransferases and cytochrome P450 expressed in Chinese hamster V79 cells. *Int. J. Cancer* **2003,** *105*, 583-592. doi: 10.1002/ijc.11143
94. Høie, A.H.; Monien, B.H.; Glatt, H.; Hjertholm, H.; Husøy, T., DNA adducts induced by food mutagen PhIP in a mouse model expressing human sulfotransferases 1A1 and 1A2. *Toxicol Lett* **2016,** *248*, 34-8. doi: 10.1016/j.toxlet.2016.02.017

95. Li, Y.; Chen, Z.; Paonessa, J.D.; Meinl, W.; Bhattacharya, A.; Glatt, H.R.; Vouros, P.; Zhang, Y., Strong impact of sulfotransferases on DNA adduct formation by 4-aminobiphenyl in bladder and liver in mice. *Cancer Med.* **2018,** *7*, 5604-5610. doi: 10.1002/cam4.1779
96. Arlt, V.M.; Meinl, W.; Florian, S.; Nagy, E.; Barta, F.; Thomann, M.; Mrizova, I.; Krais, A.M.; Liu, M.; Richards, M.; Mirza, A.; Kopka, K.; Phillips, D.H.; Glatt, H.R.; Stiborova, M.; Schmeiser, H.H., Impact of genetic modulation of SULT1A enzymes on DNA adduct formation by aristolochic acids and 3-nitrobenzanthrone. *Arch. Toxicol.* **2017,** *91*, 1957-1975. doi: 10.1007/s00204-016-1808-6
97. Sachse, B.; Meinl, W.; Glatt, H.R.; Monien, B.H., The effect of knockout of sulfotransferases 1a1 and 1d1 and of transgenic human sulfotransferases 1A1/1A2 on the formation of DNA adducts from furfuryl alcohol in mouse models. *Carcinogenesis* **2014,** *35*, 2339-45. doi: 10.1093/carcin/bgu152
98. Sachse, B.; Meinl, W.; Glatt, H.; Monien, B.H., Ethanol and 4-methylpyrazole increase DNA adduct formation of furfuryl alcohol in FVB/N wild-type mice and in mice expressing human sulfotransferases 1A1/1A2. *Carcinogenesis* **2016,** *37*, 314-319. doi: 10.1093/carcin/bgw006
99. Høie, A.H.; Monien, B.H.; Sakhi, A.K.; Glatt, H.; Hjertholm, H.; Husøy, T., Formation of DNA adducts in wild-type and transgenic mice expressing human sulfotransferases 1A1 and 1A2 after oral exposure to furfuryl alcohol. *Mutagenesis* **2015,** *30*, 643-9. doi: 10.1093/mutage/gev023
100. Høie, A.H.; Svendsen, C.; Brunborg, G.; Glatt, H.R.; Alexander, J.; Meinl, W.; Husøy, T., Genotoxicity of three food processing contaminants in transgenic mice expressing human sulfotransferases 1A1 and 1A2 as assessed by the in vivo alkaline single cell gel electrophoresis assay. *Environ. Mol. Mutagen.* **2015,** *56*, 709-714. doi: 10.1002/em.21963
